# Genome-wide analyses of chitin synthases identify horizontal gene transfers towards bacteria and allow a robust and unifying classification into fungi

**DOI:** 10.1186/s12862-016-0815-9

**Published:** 2016-11-24

**Authors:** Isabelle R. Gonçalves, Sophie Brouillet, Marie-Christine Soulié, Simonetta Gribaldo, Catherine Sirven, Noémie Charron, Martine Boccara, Mathias Choquer

**Affiliations:** 1 Univ Lyon, Université Claude Bernard Lyon 1, CNRS UMR5240, Microbiologie Adaptation et Pathogénie, Bâtiment André Lwoff, 10 rue Raphaël Dubois, F-69622 Villeurbanne, France; 2 BAYER S.A.S., Centre de Recherche de la Dargoire, F-69263 Lyon, France; 3 Sorbonne Universités, UPMC Univ Paris 06, UMR 7205 (MNHN, UPMC, CNRS, EPHE), Atelier de Bioinformatique, F-75231 Paris, Cedex 05 France; 4 Sorbonne Universités, UPMC Univ Paris 06, INRA-AgroParisTech UMR1318, F-78026 Versailles, France; 5 Institut Pasteur, Unité Biologie Moléculaire du Gène chez les Extrêmophiles, Département de Microbiologie, 25 rue du Docteur Roux, F-75015 Paris, France

**Keywords:** Chitin synthase, Bacteria, Evolution, Classification, Horizontal gene transfer, Fungi

## Abstract

**Background:**

Chitin, the second most abundant biopolymer on earth after cellulose, is found in probably all fungi, many animals (mainly invertebrates), several protists and a few algae, playing an essential role in the development of many of them. This polysaccharide is produced by type 2 glycosyltransferases, called chitin synthases (CHS). There are several contradictory classifications of CHS isoenzymes and, as regards their evolutionary history, their origin and diversity is still a matter of debate.

**Results:**

A genome-wide analysis resulted in the detection of more than eight hundred putative chitin synthases in proteomes associated with about 130 genomes. Phylogenetic analyses were performed with special care to avoid any pitfalls associated with the peculiarities of these sequences (e.g. highly variable regions, truncated or recombined sequences, long-branch attraction). This allowed us to revise and unify the fungal CHS classification and to study the evolutionary history of the CHS multigenic family. This update has the advantage of being user-friendly due to the development of a dedicated website (http://wwwabi.snv.jussieu.fr/public/CHSdb), and it includes any correspondences with previously published classifications and mutants. Concerning the evolutionary history of CHS, this family has mainly evolved via duplications and losses. However, it is likely that several horizontal gene transfers (HGT) also occurred in eukaryotic microorganisms and, even more surprisingly, in bacteria.

**Conclusions:**

This comprehensive multi-species analysis contributes to the classification of fungal CHS, in particular by optimizing its robustness, consensuality and accessibility. It also highlights the importance of HGT in the evolutionary history of CHS and describes bacterial *chs* genes for the first time. Many of the bacteria that have acquired a chitin synthase are plant pathogens (e.g. *Dickeya* spp; *Pectobacterium* spp; *Brenneria* spp; *Agrobacterium vitis* and *Pseudomonas cichorii*). Whether they are able to produce a chitin exopolysaccharide or secrete chitooligosaccharides requires further investigation.

**Electronic supplementary material:**

The online version of this article (doi:10.1186/s12862-016-0815-9) contains supplementary material, which is available to authorized users.

## Background

Chitin is a biological polymer consisting of carbohydrate molecules bonded together to form long chains of polysaccharides. Unlike starch and glycogen that are storage polysaccharides in plants and animals respectively, chitin is a structural polysaccharide organised as crystalline microfibrils and with enormous tensile strength. It contributes to the rigidity and integrity of cells, tissues or body surfaces in a wide range of organisms, protecting and giving them shape, as seen with cellulose or pectin in plant and algal cell walls. So far, the presence of this structural polysaccharide has been mainly demonstrated in the cell walls of mycota, the exoskeleton of hexapoda or crustacea, and in the radula or beak of mollusca [1], where it plays a major role in development and growth. In addition, the presence and subcellular location of chitin in invertebrate hemocytes suggests another role for this polysaccharide in the immune system of diverse animals [2]. It was generally thought that there was no chitin in vertebrates but this polymer has been described in several ray-finned fishes (Actinopterygi) [3, 4] and in some amphibians [5]. The role of endogenous chitin in the biology of these vertebrates remains elusive. Chitin has also been sporadically found in structures from a diverse range of eukaryotic microorganisms, such as the cell wall of a few chlorophyta (green microalgae), the cyst wall or lorica, of ciliophora (ciliated protozoans), the theca of choanoflagellida (flagellated protozoans), and the test or cyst wall, of amoebozoa (amoeboid protozoans) [6]. It is also present in the large family of heterokonta protists, for example in the cell wall of oomycota, the spines of diatomae and the stalk of chrysophyta [7].

Chitin is an hexosamine polymer composed of beta-(1,4)-linked linear chains of more than 5,000 N-acetylglucosamine residues that are highly cross-linked with hydrogen bonds. In insects, chitin is deposited exclusively on the apical sides of epithelial cells, facing the external environment (body surface, gut and tracheal lumen) [8]. In fungal cell walls, there is a common fibrillar core composed of branched beta-(1,3)-glucan to which chitin and other polysaccharides are covalently bound. Chitin accounts for 1-2% of the cell wall mass in yeasts and up to 30% in molds [9, 10]. Elongation of the chitin polymer is catalyzed by a highly conserved enzyme called chitin synthase, CHS (UDP-N-acetyl-D-glucosamine: chitin 4-beta-N-acetylglucosaminyl-transferase; EC 2.4.1.16). The CHS enzyme belongs to the GT2 family of processive polymerizing glycosyltransferases which includes the synthases for cellulose, callose, curdlan, mannan, hyaluronate and alginate polymers [11]. In fungi, chitin biosynthesis requires a set of multiple CHS isoenzymes that are encoded by a multigenic family. Although they share a common central catalytic domain, CHS isoenzymes from fungi and other species (metazoa, protists) can differ greatly in their N- and C-termini parts. All CHS utilize UDP-N-acetylglucosamine (UDP-GlcNAc) from the cytoplasm as a substrate and catalyze multiple transfers of the activated sugar donor to the non-reducing end of the growing chain. Multiple transmembrane domains are found in every CHS protein and these probably form a channel in the cell membrane through which linear chitin is extruded into the extracellular space as it is described for cellulose biosynthesis [12]. Nevertheless, the exact mechanism by which chitin is assembled into microfibrils and cross-linked with other components of the cell wall is still poorly understood.

The importance of *chs* genes in fungal biology has been extensively investigated by reverse genetics. Mutants have been constructed by disrupting or deleting particular *chs* genes in fungal species and no less than a hundred mutants have been made so far in more than twenty fungi (Additional file 1: Table S1). The multiplicity of *chs* genes in fungal genomes has necessitated their classification for comparative functional genomics, and fungal CHS isoenzymes have been classified into multiple divisions and classes according to protein similarities in their catalytic domain. More than 50 phylogenetic analyses have been published and differences, in the names and in the number of classes have complicated the situation (Additional file 2: Table S2). Initial attempts to classify CHS mainly involved the Ascomycota sequences (Table 1). Organising classes I, II and III into the division 1 and the classes IV and V into the division 2 was the result of working on a small number of fungal sequences [13–15]. Then, several other classes (V, VI, VII etc.) or a new division 3, were added but nomenclature was unfortunately decided heterogeneously between the different study groups [16–21]. More recently, CHS classification was extended to several basidiomycota species but by still following the same nomenclature used for Ascomycota species [22] (Table 1). Finally, a class VIII was recently proposed but it was for three completely distinct clades of putative CHS, resulting in an unusable classification [10, 23, 24]. Moreover, all these different classifications were initially based upon CHS sequences from Ascomycota and the actual classifications are not well suited to other fungi (e.g. Mucoromycotina).

**Table 1:**
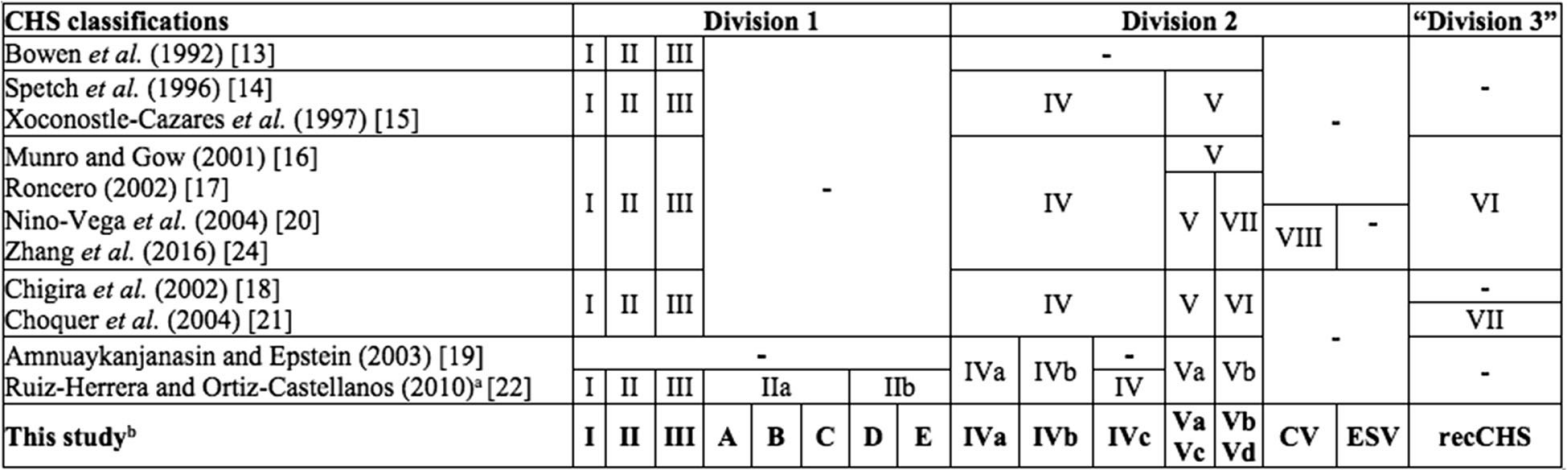
Summarized history of chitin synthase protein classifications in fungi

In bold, the chitin synthase classification issued from our study

^a^Inversion between classes IVa and IVb and inversion between classes Va and Vb were corrected in Ortiz-Castellanos and Ruiz-Herrera [110]

^b^CV: Chlorovirus-like CHS class; ESV: Ectocarpus siliculosus-like CHS class; recCHS: recombined chitin synthase

In order to update and standardize CHS classifications, we performed a comparative multi-species analysis across many sequenced and annotated genomes. A databank of CHS proteins was generated from a similarity search of CHS Pfam domains in the complete proteomes. Maximum likelihood (ML) and Bayesian phylogenetic trees were constructed to provide a global view of the whole CHS family. Applying a rigorous method of phylogenetic analysis, we organised the fungal sequences and obtained a more robust classification. We extended the study to chitin synthases from other species in order to elucidate *chs* gene evolution. In particular, this genome-wide phylogenetic analysis confirms the occurrence of multiple gene losses, duplications and horizontal gene transfers within this family of glycosyltransferases. Surprisingly, this work provides, for the first time, evidence of a chitin synthase horizontal gene transfer from eukaryota to bacterial genomes.

## Results and discussion

**Fig. 1:**
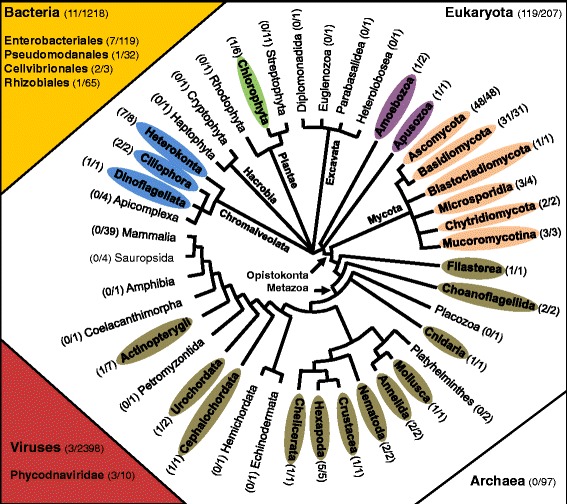
Distribution of chitin synthases among eukaryotes, bacteria and viruses. Eukaryotic species phylogeny was adapted from [100], with modifications for Hacrobia [101], Ciliophora [102], Coelacanthimorpha [103], and Amoebozoa, Apusozoa, Filasterea and Choanoflagellida [104]. Groups in which the presence of chitin synthase protein was detected are shaded in grey and written in bold. In each group, we include, in brackets, the number of species harboring chitin synthase proteins compared to the number of species analyzed. These species are listed in Additional file 3: Table S3 and Additional file 14: Table S7

**Fig. 2:**
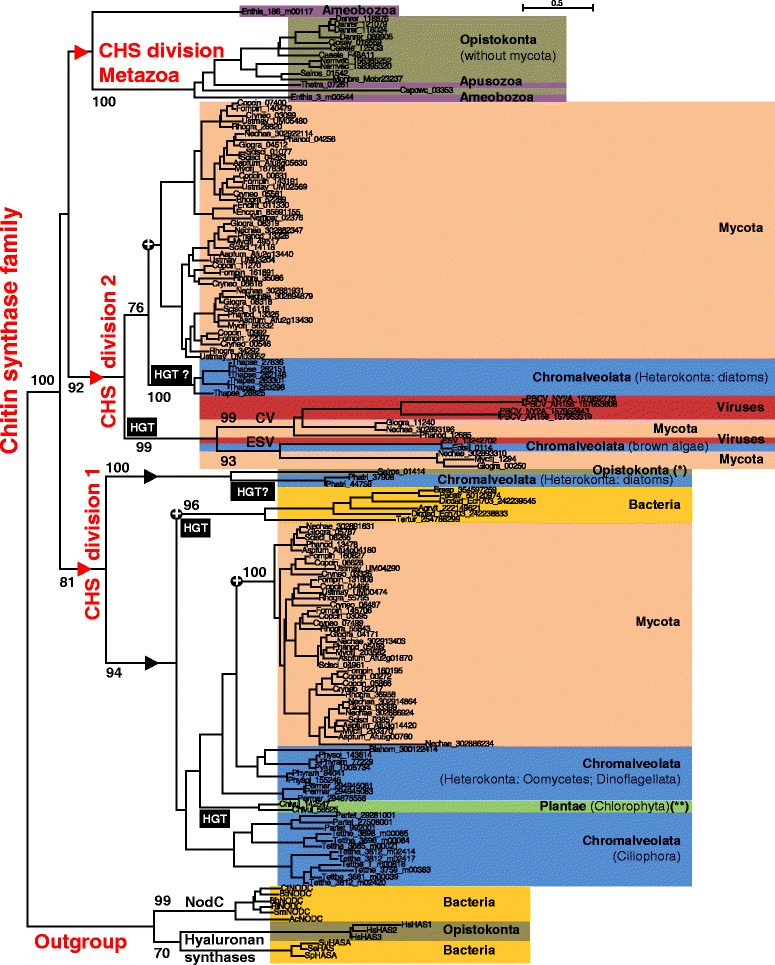
Evolution of chitin synthases (CHS). A ML phylogeny based on 222 amino acid alignment positions of 157 sequences was constructed with PhyML using NODC proteins and Hyaluronan synthases as outgroups. Bootstraps of interest ≥60 are shown above the branches. Black circles with a + correspond to root nodes of the subtrees detailed in another figure. Horizontal gene transfers (HGT) are indicated on the branches leading to transferred sequences. The Bayesian phylogenetic approach gave similar results (Additional file 15: Figure S7). The abbreviations used in sequence names are listed in Additional file 13: Table S6. (*) Acanthamoeba and Ichtyosporea sequences were also described in this group [43]. (**) *Picochlorum sp*. sequence is also present in this group [50]

*In silico* detection of putative CHS in annotated and complete proteomes enabled us to identify more than eight hundreds of CHS sequences in about 120 eukaryota species. We also found CHS sequences from a few viruses and bacteria, but no CHS could be found in archeal species (Fig. 1; Additional file 3: Table S3). Most fungal chitin synthases fall into two distinct divisions (Fig. 2).

### Update of CHS fungi classification

We observed that in Dikarya fungi, Ascomycota and Basidiomycota, up to 15 CHS-encoding genes were found in the same species (e.g. *Postia placenta;* Additional file 3: Table S3). In Ascomycota yeasts, the number of CHS is usually lower than in filamentous fungi and ranges from one (e.g. *Schizosaccharomyces pombe*) to seven (*Yarrowia lipolytica*) [25]. In Hemiascomycota yeasts, the presence of seven CHS in *Yarrowia lipolytica* and only three in *Saccharomyces cerevisiae* can only be explained by several losses in this clade (Additional file 4: Figure S1). In Mucoromycotina, Chytridiomycota and Blastocladiomycota, 15 to 38 *chs* genes were found depending on the species suggesting an even larger expansion of the CHS family in these early-branching fungal lineages (Additional file 3: Table S3). Finally, none or only one, *chs* gene was found in four Microsporidia fungal genomes (*Encephalitozoon cuniculi*, *Encephalitozoon intestinalis*, *Nematocida parisii* and *Nosema ceranae*). This characteristic seems consistent with the extreme reduction and compaction that was observed in these particular genomes [26]. The large number of detected CHS, and their diversity, provided the opportunity to establish a more robust fungal classification for this protein family. In our definition, a class means a set of CHS sequences, from different Ascomycota, Basidiomycota and/or Mucoromycotina species, which forms a well-supported group in the trees constructed with two phylogenetic approaches (ML and Bayesian). For now, we consider the genome quality and number from Chytridiomycota and Blastocladiomycota species insufficient to accurately classify these sequences. Ascomycota yeast CHS sequences were excluded from most phylogenetic analyses in order to reduce the risk of long-branch attraction (LBA) as substantial artefact was described in the phylogeny of *Saccharomyces* species [27]. LBA could have contributed to erroneously cluster together long branches, irrespective of the true relationships of sequences [28]. Such exclusion is not a problem as classification of CHS from yeasts is easy (Additional file 4: Figure S1).

#### Fungal division 1 : five new classes A to E in addition to classes I, II and III

**Fig. 3:**
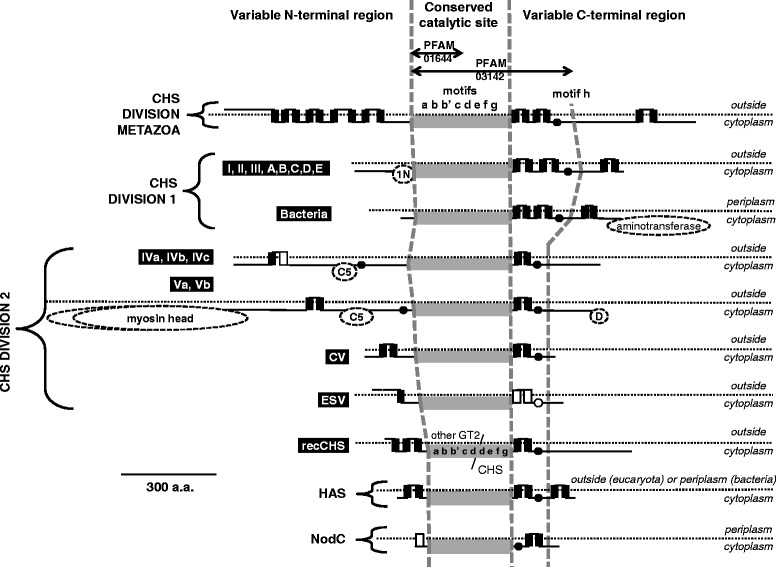
Structural features of the chitin synthase proteins. The core of CHS proteins is always composed of conserved motifs named a-h and playing a role in the active site of the enzyme (Additional file 7: Figure S3). The domains related to chitin synthase used for their detection, Chitin_synth_1 (PF01644) and Chitin_synth_2 (PF03142), correspond to the a-c and a-h regions respectively. Other domains from the Pfam library, 1N (Chitin_synth_1N; PF08407), Aminotransferase (DegT_DnrJ_EryC1; PF01041), Myosin head (Myosin_head; P00063), C5 (Cyt-b5; PF00173) and D (DEK_C; PF08766) were often detected in some CHS clades and are indicated by dashed boxes. The length variation of the myosin head domain in the clade V is represented by a dashed line in the dashed box of this domain. N-terminal and C-terminal regions of CHS are variable and transmembrane segments detected in almost all the sequences of one clade are shown with black solid squares. The less frequent additional segments are shown with white solid squares. Predicted outside or cytoplasmic localizations of eukaryota CHS protein segments are indicated thanks to a dotted line (or periplasmic and cytoplasmic localizations for bacterial CHS and HAS). Cytoplasmic localization of the conserved motifs a-h in homologous GT2 glycosyltransferases was confirmed with LacZ, PhoA and/or GFP reporter fusions [105–109]. According to these studies, some predicted transmembrane domains are, in fact, putative membrane domains that do not cross the membrane (black circles or white circles for the less frequent ones). The distant motif h might interact with the other conserved motifs as its cytoplasmic localization was confirmed

**Fig. 4:**
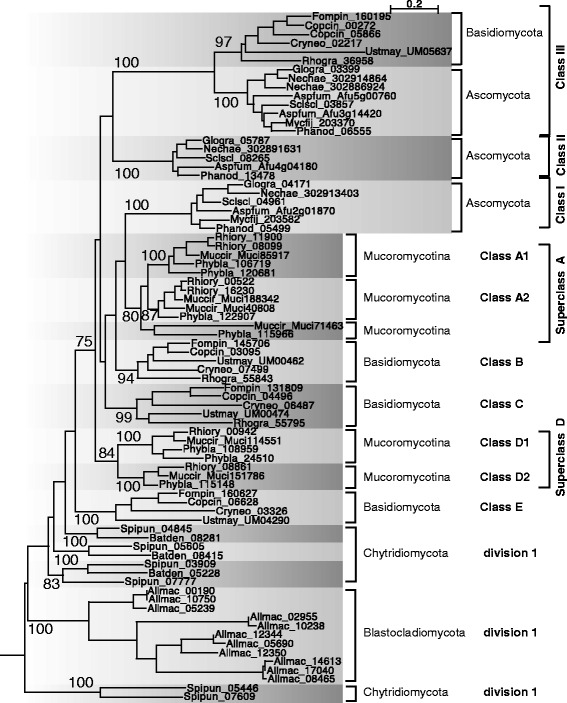
Evolution of fungal CHS belonging to division 1. A ML phylogeny, based on 489 amino acid alignment positions of 78 sequences, was constructed with PhyML. The root was placed according to the phylogeny in the Additional file 16: File S2. The Bayesian phylogenetic approach gave similar results (Additional file 17: Figure S8)

Fungal division 1 consists mainly of protein sequences with an N-terminal Chitin_synth_1N domain (PF08407), a conserved catalytic site and 6 predicted transmembrane domains in the C-terminal region (Fig. 3). In this division, different fungal lineage-specific classes were found with only one, corresponding to class III, which was common to Ascomycota and Basidimycota (Fig. 4). Previous attempts to group the classes A to E mainly with Ascomycota classes I or II resulted in different classifications as these groups were not well supported by the phylogenies (see references in Additional file 2: Table S2). A possible explanation for the observed phylogeny is that several duplications arose for this division at an early stage in fungal evolution and, thereafter, different gene losses might have occurred in the different lineages. More than forty fungal mutants impaired in a division 1 *chs* gene were generated in Ascomycota species and a few in Basidiomycota species (see references in Additional file 1: Table S1). Some differences in CHS activity in vitro, chitin content and conidiation were observed for *chs* mutants impaired in Ascomycota classes I or II. These classes probably have redundant roles as the single corresponding mutants were not affected in their growth and morphology. On the contrary, class III CHS seems to play a crucial role in the hyphal tip growth as several class III mutants were strongly affected in their morphology, with reduced growth resulting in small colonies and abnormal highly-branched hyphae (Additional file 1: Table S1). This class was lost early on during Saccharomycotina yeast evolution, as only *Yarrowia lipolytica* possess a class III *chs* gene (Additional file 4: Figure S1). Conversely, this class was expanded in some Pezizomycotina species as already mentioned for *Fusarium sp.* and *Aspergillus sp.* [23]. Some duplicated copies are fast evolving sequences, which contrasts with the strong sequence conservation observed in this class, and they might have acquired different roles (Additional file 5: Figure S2).

#### Fungal division 2 : two superclasses IV and V

**Fig. 5:**
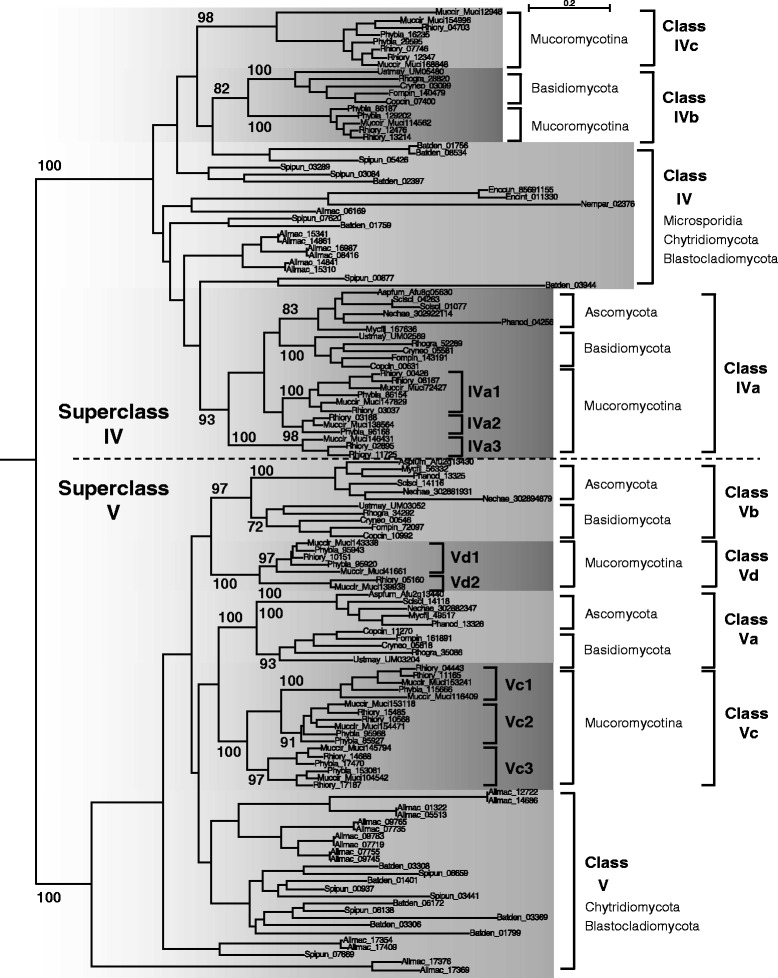
Evolution of fungal CHS belonging to division 2. A ML phylogeny, based on 485 amino acid alignment positions of 131 sequences, was constructed with PhyML. The root was placed according to the phylogeny in the Additional file 18: File S3. The Bayesian phylogenetic approach gave similar results (Additional file 19: Figure S9)

Fungal division 2 is clearly divided into two monophyletic superclasses IV and V (Fig. 5). These are composed of protein sequences which contain, in addition to the chitin synthase catalytic site, one or two predicted transmembrane domains in the N-ter and C-ter regions plus a cytochrome-b5-like domain (PF00173) in the N-ter region, which has a proposed role as a binding site for lipid ligands [29] (Fig. 3). Sequences of the superclass V often have two additional domains: a myosin motor domain (PF00063) fused to their N-terminus extremity, involved in intracellular trafficking of CHS and site specificity of chitin secretion [30] (Fig. 3), and a DEK-C domain (PF08766), of unknown function, at their C-terminus. Each superclass was divided into several classes, most of which are conserved between Ascomycota, Basidiomycota and Mucoromycotina (Fig. 5). The class IVb was lost in Ascomycota and the ancestor of most Hemiascomycota yeasts lost all division 2 classes except the class IVa (Additional file 4: Figure S1). The class IVc was only found in Mucoromycotina, where an expansion of classes IVa, Va and Vb was also observed. Proteins from superclasses IV and V were also detected in the proteomes of Chytridiomycota and Blastocladiomycota whereas Microsporidia proteomes only contained one member of the superclass IV. Despite the fact that the superclass IV was found in the largest number of fungal species, the corresponding mutants do not usually exhibit any apparent phenotypic change compared to the wild-type strain (Additional file 1: Table S1). By contrast, mutants of class Va or Vb are usually strongly affected in their morphology (swelling, baloon formation, intrahyphal hyphae), in the response to cell wall stresses and in virulence [31] (Additional file 1: Table S1).

#### Fungal division 2: two virus-like classes

In fungal division 2, in addition to the two superclasses IV and V, two others classes were found (Fig. 2). These fungal classes, that we have called ESV (*Ectocarpus siliculosus* Virus-like CHS) and CV (Chloroviruses-like CHS), were found in proteomes of some Ascomycota (Sordariomycetes, Dothideomycetes and Eurotiomycetes), some Basidiomycota (Ustilaginomycetes only possessing a ESV CHS), a Chytridiomycota (only possessing a CV CHS) and some Phycodnaviridae giant algal-viruses (Additional file 6: Table S4). The CV class was recently proposed as a new class VIII [24] but we do not recommend this denomination as class VIII is used for other distinct groups of putative CHS [10, 23] (Additional file 2: Table S2). Independent horizontal transfers of these *chs* genes with their neighboring genes, in fungal genomes from chloroviruses and phaeoviruses respectively, have already been suggested [32]. In addition to the strong similarity between CV and ESV viral and fungal sequences, the corresponding genes are clustered on genomes with an UDP-N-acetylglucosamine 6-dehydrogenase gene (UNGD). This two-gene cluster, conserved in some fungal species and in viruses of distantly related algae, suggests that these two genes were probably transmitted together and that this gene pair might work in concert : UNGD family enzymes provide precursors for glycosyltransferase enzymes [32]. Moreover, the fungal and viral CV genes are also colocated with a polysaccharide deacetylase. These genetic material exchanges between fungi and algal-viruses are perplexing. However, as exemplified by the 600 million year old lichen-fossils, interactions betwen fungi and algae have long existed [33]. Furthermore, gene transfers from the ancestor of Dothideo/Sordariomycetes to the ancestor of the terrestrial alga *Trebouxia decolorans* have also been proposed [34]. There is currently no evidence for the functionality of the fungal proteins encoded by the genes from the ESV class. However, the CV class genes of *Glomerella graminicola* and *Gibberella zeae* were found to be differentially expressed during plant infection [23] and a deletion mutant in a CV class *chs* gene was recently described in *Fusarium graminearum* (called *Fgchs8* gene) [24]. Disruption of this gene resulted in a reduced accumulation of chitin, decreased CHS activity, sensitivity to SDS and reduced pathogenicity. It has been suggested that the *Fgchs8* gene is required for cell wall development in *F. graminearum*. The *chs* genes from the CV and ESV classes were duplicated in some Ascomycota lineages and they were lost in others [32] (Additional file 6: Table S4). They also show higher evolutionary rates than other fungal CHS (Fig. 2; Additional file 5: Figure S2). The virus containing a *chs* gene from the ESV class is, more precisely, a lysogenic phaeovirus. DNA from this virus, including the ESV *chs* gene, is integrated into the genome of the pluricellular brown alga *Ectocarpus Siliculosus* [35]. This integrated viral ESV *chs* gene is transcriptionally silent in the algae and is probably not functional [36]. Viruses with at least one *chs* gene from the CV class include some chloroviruses infecting the unicellular green alga *Chlorella* with a lytic infection style (e.g. Paramecium Bursaria Chlorella viruses). Some chloroviruses form chitin on the surface of infected cells which might protect virus-infected algae from uptake by other organisms [37]. Indeed, the heterologous expression of the chlorovirus CVK2 *chs* gene was performed in *E. coli* leading to the production of fibers by the bacterium [38].

#### Outside fungal divisions : a class of CHS probably emerging from a recombination event

In our analysis, a complete CHS class, previously called VI, VII (Table 1; Additional file 2: Table S2) or sometimes division 3 [39], was treated separately. We suspect a recombination event of being at the origin of this class (Fig. 3). Associated with a reduced taxonomic distribution in fungi, including some Ascomycota groups (Sordariomycetes, Dothideomycetes, Eurotiomycetes and Leotiomycetes) and a Mucoromycotina species (Additional file 6: Table S4), this class was also recently detected in a chromalveolate (Rhizaria), *Plasmodiophora brassicae*, an obligate biotrophic pathogen of crucifers [40]. In this study, we suggest that the corresponding protein sequences have characteristics associated with a rearrangement in their ancestor: a duplication of the QXXXY motif (motif d in Fig. 3 and Additional file 7: Figure S3) and a phylogenetic signal which seems to differ in the N- and C-termini sequence fragments located on either side of this duplication (Additional file 8: Figure S4). Indeed, while the C-terminal region appears to be similar to that in other CHS (Additional file 8: Figure S4A), the N-terminal region is closer to hyaluronan synthase proteins in the phylogenetic tree (Additional file 8: Figure S4B) and shares the same organization of transmembrane domains (Fig. 3). This observation could be explained by an ancient recombination between the ancestor’s sequences of two glycosytransferase family 2 proteins. An alternative explanation, that we can not completely exclude, is that the ancestor of this class underwent a period of an accelerated rate of evolution which blurred the phylogenetic signal. In both cases (recombination or transient high evolutionary rate), we excluded them from phylogenetic analyses as they might have provoked artefactual groups due to long-branch attractions. HGTs are probably at the origin of this CHS class in one Mucoromycotina (*Mortierella verticillata*), one Chytridiomycota (*Batrachochytrium dendrobatidis)* and one chromalveolata (*Plasmodiophora brassicae)* (Additional file 6: Table S4). In Ascomycota groups, where this class is present, the corresponding genes are probably functional as they are strictly fixed in one well-conserved copy (Additional file 3: Table S3; Additional file 5: Figure S2). However, their chitin synthase activity has not yet been proved so we recommend using the term recCHS (“recombined” CHS) for these sequences. Few fungal deletion mutants were obtained for this class and their phenotypes are divergent. RecCHS orthologs have probably evolved with different roles in these fungi during growth and development [41, 42].

### Origin of CHS in eukaryotes

The phylogeny was obtained with a protein sequences sample representative of the entire set of chitin synthases detected in this study (Fig. 2). At least three chitin synthase genes were present in the ancestor genome of Opistokonta: one ancestral *chs* for the Metazoa division, one for the division 2 and one for the division 1 (see red triangles in Fig. 2). A previous study suggested four ancestral *chs* genes in the Last Opisthokonta Common Ancestor (LOCA) [43] but we propose that two of them belong to division 1 and they may have diverged from a common ancestral sequence in LOCA (see black triangles in Fig. 2). The chitin synthase genes probably appeared earlier, given the basal positions in the Metazoa division of one sequence of the Amoebozoa *Entamoeba histolytica* and the CHS of the apusomonad *Thecamonas trahens*. The distribution of taxa possessing a *chs* gene in Metazoa indicates that *chs* genes were lost several times independently in this phylum (Fig. 1; in Platyhelminthes, in Hemichordata, in some Actinopterygii etc.). On the other hand, family expansion occurred in some species, such as in the gastropod *Lottia gigantea* [44] and the amphioxus *Branchiostoma floridae* [45]. It is noteworthy that a second CHS sequence from *Entamoeba histolytica* did not significantly group with any of the chitin synthase divisions, which raises the question of its origin. However, heterologous expression of this protein in the yeast *Saccharomyces cerevisiae* confirmed its CHS activity [46]. The presence of chitin synthases belonging to different divisions in diverse chromalveolates, such as ciliates, diatoms, oomycetes and other protists, might be the result of a deeper evolutionary origin of CHS in the Last Common Eukaryotic Ancestor [40, 47] (Figs. 1 and 2; Additional file 3: Table S3). However, an alternative plausible model for the origin of chromalveolate CHS could imply several independent horizontal gene transfers at different times during the chromalveolate evolutionary history [48], as we suggested for the *chs* of the rhizaria *Plasmodiophora brassicae* (see above in Update of CHS fungi classification). Diatom *chs* genes from *Thalassiosira pseudonana* (division 2) and *Phaeodactylum tricornutum* (division 1), were probably acquired by an HGT from Opistokonta (fungi or metazoa; Fig. 2).

Other HGT must have occurred to explain the actual taxonomic distribution of *chs* genes and their sequence diversity. If the division 1 *chs* of the green algae (Trebouxiophyceae*) Chlorella* [49] (Fig. 2) and *Picochlorum* [50] were vertically inherited from the Plantae ancestor, it would imply that these genes were independently lost in many lineages of the plantae. As mentioned for the CV and ESV classes of division 2 (Update of CHS fungi classification), *chs* gene exchanges might have occurred between algae-related viruses, including chloroviruses and fungi. However, the *Chlorella* CHS belongs to division 1 and seems to resemble oomycete and ciliate CHS more than fungal ones (Fig. 2). Therefore, a possible source of the transfer could be a ciliate living in symbiosis with a green alga, such as *Paramecium bursaria*.

Hence, the evolutionary history of chitin synthase genes suggests different independent horizontal gene transfers among diverse eukaryotic microorganisms (Fig. 2). While viruses are known to be gene transfer agents, membrane vesicles, which are not impaired by receptor recognition in the way that viruses are, could be large spectrum transducing agents [51–53].

### Bacterial chitin synthase genes

**Fig. 6:**
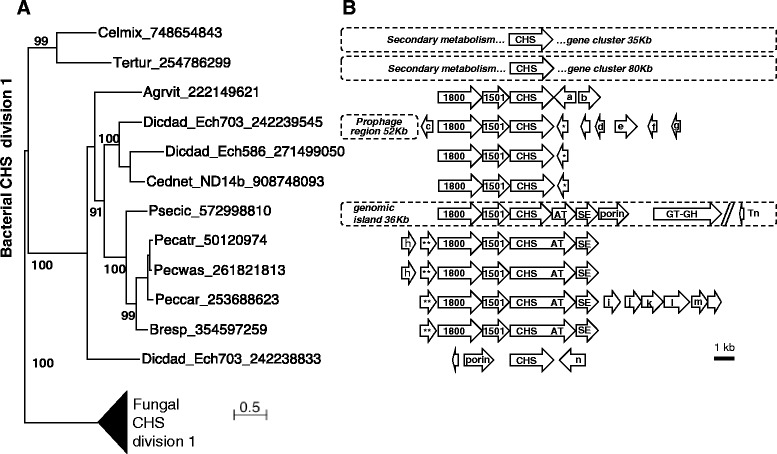
Phylogeny and genomic context of bacterial chitin synthases. **a** Phylogenetic relationships of bacterial chitin synthases. The ML tree is shown with numbers above the branches, indicating support in bootstrap analyses (100 replicates). The tree was rooted with division 1 fungal CHS from *A. fumigatus* and *U. maydis*. The Bayesian phylogenetic approach gave the exact same topology (Additional file 20: Figure S10). **b** Gene organization of each variable region containing chitin synthase. The limits of regions were obtained by comparison with ortholog regions in proximal species without the *chs* gene. Each represented region is aligned with the corresponding CHS in the phylogenetic tree. CHS in arrows is the chitin synthase domain and AT is the aminotransferase domain. 1800 and 1501: proteins with a domain of unknown function, DUF1800 (PF08811) and DUF1501 (PF07394) respectively; SE: sugar epimerase; a: transcriptional regulator with a sugar-binding domain; b: 2,4-dihydroxyhept-2-ene-1,7-dioic acid aldolase; c: OsmC family protein; d: DUF465; e: lipid A biosynthesis lauroyl (or palmitoleoyl) acyltransferase; f: cold-shock protein; g: DUF2511; h: efflux protein; i: gluconate-5-dehydrogenase; j: taurine dioxygenase; k: sulfotransferase NodH; l: aminotransferase; m: hydroxylase; and n: AraC family transcriptional regulator. The dotted rectangles correspond to regions detected as a prophage, a secondary metabolism cluster or a genomic island. Empty arrows and those with stars represent hypothetical proteins. Among them, groups of arrows with the same number of stars are orthologs

The analysis of bacterial proteomes gave unexpected results as it revealed a dozen of bacteria possessing at least one division 1 CHS whereas no bacterial CHS had been previously described (Figs. 1, 2 and 6; Additional file 3: Table S3). These bacteria correspond mainly to Gammaproteobacteria (7 Enterobacteriaceae, 2 Cellvibrionaceae and 1 Pseudomonadaceae) but there is also an Alphaproteobacteria (Rhizobiaceae). Finally, one *chs* gene was detected in the genome of the Cyanobacteria *Tolypothrix campylonemoides* (NCBI Reference Sequence: NZ_JXB01000008) but it was not included as it is split by a transposase insertion and is probably not functional. The well supported monophyletic group, formed by the corresponding CHS proteins, suggests a unique transfer of a eukaryotic division 1 *chs* gene to a bacterial genome and multiple HGT might have occurred then between bacterial species. Several cases of the lateral transfer of genes, protein domain-encoding fragments or repeat elements have been described, from animals to bacteria (reviewed in [54–56]) and from Plantae to bacteria [57–59]. However, the detected eukaryote to bacteria transfers are fewer than the bacteria to eukaryote ones [54, 60] for the following possible reasons: (i) the barrier formed by spliceosomal introns in eukaryotic genes [54, 61]; (ii) the smaller population size of eukaryotes which, therefore, offers a reduced pool of potential donors [61]; and (iii) the small number of eukaryotic genes that might present a selective advantage for bacteria and which could, thus, persist in their genome after being transferred [62]. Interestingly, the family 14 of carbohydrate-binding modules (CBM14s), which are short chitin-binding modules predominantly found in animal and fungal genomes, were also detected in seven bacterial genomes as putative HGT [63]. However, the bacterial species involved were different from those harboring chitin synthase genes. Most of the bacterial *chs* genes were co-localized with a set of co-oriented genes, in a classical bacterial operon organization (Fig. 6) including a sugar epimerase and hypothetical protein encoding-genes. This unusual pattern of gene order conservation between distantly related bacteria suggest that the corresponding genes were transferred and maintained together, possibly because they participate in a common function in these bacteria. Noticeably, two of the hypothetical proteins possess bacterial domains of unknown functions DUF1800 and DUF1501, that are predicted to be part of the same operon in OperonDB [64, 65]. Enterobacteriales species (*Brenneria*, *Pectobacterium* and *Dickeya*) clearly acquired these DUF- and CHS-encoding genes during the same transfer because close enterobacteriales relatives without *chs* have neither the DUF1501- nor the DUF1800-encoding genes (Additional file 9: Table S5). The alphaproteobacteria *Agrobacterium vitis* has two pairs of DUF1501-DUF1800 encoding genes organized in tandem. Phylogenetic trees revealed that the pair of proteins encoded by the genes co-localized with the *chs* are more closely related to their homologs in gammaproteobacteria than to their homologs in alphaproteobacteria (Additional file 10: Figure S5). Hence, these DUF-encoding genes were also transferred with the *chs* in *A. vitis*. Some transfers seem to be recent as the corresponding *chs* belongs to a genomic region with a different G + C content, compared with the neighboring genomic context (Additional file 11: Figure S6). This is the case for one of the two Dickeya_dadantii_Ech703 *chs* genes (second HGT of a *chs* in this genome) and the *chs* of *Cedecea neteri* and *Pseudomonas cichorii*, which are localized in regions with a lower G + C content. The secondary metabolism gene cluster, containing one *chs* in *Teredinibacter turnereae,* also appeared with a different G + C composition higher than the neighboring genomic context. Some bacterial chitin synthase genes have evolved additional features that differentiate them from eukaryotic division 1 CHS (Fig. 6). First, they have a short N-terminal region and they lack the Eukaryota Chitin_synt_1N domain (PF08407) (Fig. 3). Secondly, an accretion of an aminotransferase domain probably occurred in the ancestor of *Pectobacterium* and *Brenneria* (Fig. 3).

In a large variety of bacteria, type 2 glycosyltransferases (GT2) homologous to chitin synthases have been described. These transmembrane enzymes are localized at the inner membrane of Gram negative bacteria and synthesize different exopolysaccharides (EPS) into the periplasm. These EPS are made of cellulose, curdlan, alginate or hyaluronic acid [66]. In *Pseudomonas aeruginosa*, secretion of alginate through the peptidoglycan and the outer membrane is ensured by an envelope-spanning multiprotein complex including channel proteins [67]. We found that some bacterial *chs* genes are colocated with porin or efflux protein-encoding genes (Fig. 6). It is possible that these bacterial *chs* genes, and their neighbouring transporter encoding-genes, are involved in the production and secretion of an EPS made of chitin.

EPS are matrix components of bacterial biofilms which are known to play a major role in pathogenic or symbiotic interactions between bacteria and animals or plants [68]. It is noticeable that, among bacteria in which a *chs* gene was found, *Pseudomonas cichorii* and *Agrobacterium vitis* are plant-pathogens and secrete an EPS made of alginate and curdlan, respectively [69, 70]. Indeed, *A. vitis* is able to build biofilms on abiotic as well as on plant root surfaces, probably due to its EPS [71]. Other bacterial species displaying a *chs* gene are also plant pathogens: a *Brenneria* spp.*,* three *Pectobacterium* species and two *Dickeya dadantii* isolates. The later two bacterial isolates, *D. dadantii* Ech586 and *D. dadantii* Ech703, should be reclassified as two different species, *Dickeya zeae* Ech586 and *Dickeya paradisiaca* Ech703, respectively [72]. It may be that the bacterial chitin synthase plays a role in the parasitic interaction between these bacteria and their plant hosts.

CHS activities in fungi or metazoa are usually described as producing long crystalline chains of chitin (fibers) but it is possible that a bacterial CHS secretes small soluble chitosaccharides, or eventually chitooligosaccharides (COS), instead. Indeed, the phytopathogenic oomycete *Aphanomyces euteiches* does not secrete chitin fibers although two *chs* genes are present in this species [73]. However, the encoded CHS activities of *A. euteiches* are thought to be active because a small soluble and noncrystalline glucan-chitosaccharide was detected in its cell wall [74]. Interestingly, one of the two *chs* genes in *D. dadantii* Ech703 and the *chs* gene of *Pseudomonas cichorii* are co-located and have the same orientation as a putative chitoporin-encoding gene (Fig. 6). In the marine bacterium *Vibrio harveyi*, chitoporin is a transporter of COS through the outer membrane [75]. COS are known to play a key role as signal molecules in multiple plant-microbe interactions. In parasitic interactions between plants and fungi, COS are released from the digestion of the fungal cell wall by secreted plant chitinases. The resulting COS are then recognized by plant receptors as Microbial Associated Molecular Patterns (MAMP) and they induce plant immunity [76]. In symbiotic interactions, modified COS, called lipochitooligosaccharides (LCOs), which include Myc and Nod factors, modulate plant host immunity. Myc factors are produced and secreted by arbuscular mycorrhizal fungi but the enzymes responsible for their biosynthesis have not yet been identified [77]. Nod factors are synthesized by NodC, a bacterial GT2 homologous to CHS, and they induce nodule formation during a symbiotic interaction between Rhizobiacea (nitrogen-fixing bacteria) and leguminous host plants [78]. Whether bacterial *chs* genes are able to synthesize COS implicated in plant interactions is still uncertain.

Several bacterial *chs* genes are fused with a putative aminotransferase domain and they are colocated with a putative sugar epimerase-encoding gene (Fig. 6). These two additional functions could be associated with modifications of chitin or COS. A gene of unknown functions, and composed of the DUF1501 domain, was also found colocated with several bacterial *chs*. The encoded DUF1501-containing protein carries a twin-arginine motif which would imply that it is exported by the twin-arginine translocation (Tat) pathway (http://www.compgen.org/tools/PRED-TAT/ [79]). The bacterial Tat system allows folded proteins to be moved across membranes without significant ion leakage [80]. The DUF1501 domain has also been described as forming part of a conserved machinery in compartimentalized species from the Planctomycetes, Verrucomicrobia and Chlamydiae (PVC) super-phylum [81]. These informations about DUF1501 proteins are hardly connectable with the biological role of known CHS. However, our hypothesis is that DUF1501, DUF1800 and CHS proteins might have a functional link in bacteria as they share a common conserved operon organization. It would be very interesting to study the function of bacterial CHS and also of the proteins that seem associated to them. Finally, in the particular cases of *Teredinibacter turnerae* and *Cellvibrio mixtus* subsp. *mixtus*, the *chs* genes are localized in the middle of secondary metabolism gene clusters (80 Kb and 35 Kb, respectively) and they could be involved in the production of a bioactive molecule [82].

## Conclusions

Bacterial chitin synthase genes constitute a new example of genes acquired by a bacterium via horizontal transfer from a eukaryotic donor. The chitin synthase activity of the bacterial CHS and the possible selective advantage for the corresponding bacteria, often implied in plant interactions, needs further investigation. CHS-encoding genes have also been transferred between eukaryotic microorganisms. We recommend avoiding the use of this multigenic family to elucidate the phylogenetic relationships between the different eukaryotic species, especially since many duplications and losses are also observed in different lineages.

Fungal CHS are, unfortunately, not an exception and the difficulty of classifying these sequences has led to discordant classifications. We took advantage of the current study to determine which can be robustly classified and then to construct the most consensual classification possible. To facilitate the use of this new classification, any information that corresponds with the previously published versions is provided (Additional file 2: Table S2), together with the databank, in fasta format, of all the classified CHS sequences (Additional file 12: File S1). A website also permits blast queries to the databank (http://wwwabi.snv.jussieu.fr/public/CHSdb). This facilitates a search for the class of a previously identified CHS, even if the corresponding accession number has changed (as is often the case for fungal sequences). It is also an aid for the classification of CHS from species closely related to those analyzed.

## Methods

### Data collection

The 208 eukaryotic complete proteomes were downloaded from different databases (Additional file 13: Table S6). The main sources were: Ensembl (56 species; http://www.ensembl.org/), the DOE Joint Genome Institute (53 species; http://www.jgi.doe.gov/), the National Center for Biotechnology Information (31 species; http://www.ncbi.nlm.nih.gov/), and the Broad Institute of MIT and Harvard (29 species; http://www.broadinstitute.org/). The tested proteomes of 1218 bacteria, 97 archaea and 2398 viruses were also downloaded from the NCBI (Additional file 14: Table S7). The hyaluronan synthase and nodulation NodC protein sequences employed in this study were those used in [21] and [83].

### Identification of chitin synthases

CHS protein sequences share several motifs (Fig. 3; Additional file 7: Figure S3), spanning the catalytic site and the C-terminal part of these enzymes, and two major related chitin synthase Pfam domains [84], Chitin_synth_1 (PF01644) and Chitin_synth_2 (PF03142). To identify the CHS sequences, similarity searches were first performed with these two domains in complete proteomes (see Methods – Data collection) using the RPS-BLAST program [85], with an E-value cutoff of 1e-05. This cutoff was judged sufficiently high to detect all chitin synthase proteins as some hits corresponded to other glycosyltransferase proteins of the GT2 family. Indeed, unlike the Chitin_synth_1 domain which is very specific to division 1 CHS sequences, the Chitin_synth_2 domain could be found in dozens of glycosyl transferase sequences that were not chitin synthases (e.g. hyaluronan synthases, cellulose synthases etc.). To eliminate these non-CHS sequences, phylogenetic trees were performed (see Methods – Sequences analysis) and only proteins forming a clear monophyletic group with known chitin synthase divisions, using hyaluronan synthase and nodulation NodC protein sequences as the outgroups, were considered as chitin synthase proteins. The presence of conserved motifs essential for enzymatic activity (D, D, D, QXXRW) was also checked as it guarantees that the proteins are potentially functional and could endow a chitin synthase activity. Proteins that do not possess these motifs were, thus, considered as dubious and disqualified from our study. For example, a recently proposed class VIII [10] was not retained due to the absence of the essential motif QXXRW.

Some CHS classes showed reduced taxonomic distribution (recCHS, ESV, CV, Bacterial CHS etc.). To gain a better understanding of the origin of these sequences, it was important to have a more precise idea of the species that possess the corresponding genes. In these cases, the analysis was completed by a BLASTP search, carried out at the NCBI, using the non-redundant protein sequences (*nr*) database.

### Sequence analysis

All phylogenetic analyses were performed using the following procedure. First, amino acid sequences were aligned using MAFFT with the E-INSI algorithm and default settings [86]. Next, regions in the resulting multiple sequence alignments that were suitable for phylogenetic inference were selected using BMGE [87]. The BLOSUM60 and BLOSUM30 matrices were used, respectively, for alignments with sequences from a single division and alignments containing sequences from different divisions. This step removed any ambiguously aligned or highly variable regions in order to improve the overall performance of the phylogenetic reconstructions. Thirdly, phylogenetic inferences were obtained with two approaches. ML trees were constructed with PhyML 3.0 [88] using the following parameters: the LG model, with empirical amino acid frequencies, an estimated proportion of invariable sites, subtree pruning and regrafting (SPR) and five random starting trees added to the standard BioNJ starting tree. The support of the data for each internal branch of the phylogenies was estimated using non-parametric bootstraps, with 100 replicates. Bayesian inferences were obtained with MrBayes v3.2.6 [89] with a fixed WAG model of amino acid substitution and a gamma correction (four discrete categories plus a proportion of invariant sites). The program was run with four chains for 100 000 generations and trees were sampled every 100 generations. To construct the consensus tree, the first 250 trees were discarded “burnin” and posterior probabilities were used as support for internal branches. NJplot [90] and FigTree [91] were used for outputting ML and Bayesian trees respectively.

Hyaluronan synthase (HAS) and NodC sequences were used as the outgroup for the global CHS tree (Fig. 2) as they are distinct type 2 glycosyltransferases. All CHS sequences from the databank were classified using phylogenies. However, only a part of them was used in the phylogenetic trees presented here, so that the trees remain readable. They correspond to the CHS of a sample of species chosen to maximised the preservation of the global CHS tree topology observed with all the sequences.

The domains were annotated by searching protein sequences against the Pfam library of HMMs with pfam_scan.pl [92]. The transmembrane helices in proteins were predicted with TMHMM 2.0 [93].

### Genomic context analysis

The genomic context of bacterial chitin synthases was analyzed with multiple genome alignments using Mauve 2.3.1 [94]. Gene organization around the chitin synthase encoding-genes was compared with that of ortholog regions in genome(s) from proximate species lacking the chitin synthase gene. Hence, four groups of genomes were compared. First, the Pectobacterium group comprised *Pectobacterium carotovorum subsp. carotovorum WPP14*, *P. carotovorum subsp. brasiliensis PBR1692*, *Brenneria sp. EniD312, P. wasabiae WPP163*, *P. carotovorum subsp. carotovorum PC1* and *P. atrosepticum SCRI1043*. Second, a *Dickeya* group comprised *Dickeya dadantii 3937*, *D. dadantii Ech586*, *D. dadantii Ech703* and *D. zeae Ech1591*. Third, an *Agrobacterium* group was composed of *Agrobacterium vitis S4* and *Agrobacterium radiobacter K84*. Finally, a Pseudomonas group comprised *P. cichorii JBC1*, *P. syringae pv. tomato DC300*, *P. syringae pv. syringae B728a* and *P. syringae pv. phaseolicola 1448A*. In each case, this allowed us to localize the limits between the variable region containing the chitin synthase coding gene and the core surrounding regions shared by all proximate species (syntenic shared blocks). Predictions concerning genomic islands were retrieved from IslandViewer [95] and predicted prophages were sourced from Prophinder [96]. A G + C content analysis was performed with overlapping sliding windows of 1000 bp at a step of 30 bp using JaDis [97].

## Additional files

Additional file 1: Table S1. Classification of fungal chitin synthase single mutants and comparison of their phenotype (1986–2016). In the first tab, phenotypes of mutants are summarized. In the second tab, phenotypes of mutants are detailed. In the third tab are the references of the single mutants publications. (XLSX 59 kb)
Additional file 2: Table S2. Correspondence between the published chitin synthase classifications (1992–2016) (first tab) and their references (second tab). (XLSX 29 kb)
Additional file 3: Table S3. Account of all chitin synthase encoding sequences in the genomes of species included in this study. (XLSX 81 kb)
Additional file 4: Figure S1. Phylogeny of Hemiascomycota yeast CHS. A ML phylogeny based on 404 amino acid alignment positions of 42 sequences was constructed with PhyML. Bootstraps of interest and ≥60 are shown above the branches. (PDF 49 kb)
Additional file 5: Figure S2. Nonsynonymous substitution rates (dN) estimated for pairwise comparisons between ascomycota *chs* from the different classes. Comparisons were restricted to a region spanning the catalytic domain from the c motif to the g motif (Fig. 3 and Additional file 7: Figure S3). The PAML [98] codeml program was used to estimate dN with the codon substitution model of Goldman and Yang [99]. (PDF 47 kb)
Additional file 6: Table S4. Account of species displaying recCHS, ESV or CV chitin synthases (tab 1) and account detail of recCHS, ESV and CV sequences in these species (tab 2). (XLSX 15 kb)
Additional file 7: Figure S3. Eight conserved signatures in chitin synthases (adapted from [21]). (PDF 90 kb)
Additional file 8: Figure S4. Phylogenies of CHS N- and C-termini regions. The N-terminal region, used for phylogeny, ends before the motif d of the catalytic domain and the C-terminal region starts after this motif (Fig. 3 and Additional file 7: Figure S3) The two corresponding alignments were obtained by extracting these regions from the alignement obtained with the full sequences. They were then filtered with BMGE prior to the tree reconstruction analysis. This resulted in 72 amino acid alignment positions of 119 CHS N-terminal region sequences and 148 amino acid alignment positions of 120 CHS C-terminal region sequences. Phylogenetic trees were constructed with PhyML (A-B) and MrBayes (C-D) Bootstraps ≥60 (PhyML) and posterior probabilities (MrBayes) of interest are shown above the branches. (PDF 133 kb)
Additional file 9: Table S5. Co-occurrence of DUF1501, DUF1800 and CHS encoding genes in bacterial genomes. (DOCX 60 kb)
Additional file 10: Figure S5. Phylogenies of DUF1800 and DUF1501 bearing proteins. ML phylogenies based on (A) 383 amino acid alignment positions of 14 DUF1800 bearing sequences and (B) 335 amino acid alignment positions of 14 DUF1501 bearing sequences were constructed with PhyML. Bootstraps of interest and ≥60 are shown above the branches. (PDF 51 kb)
Additional file 11: Figure S6. G + C content variation around the recently laterally transferred chitin synthase genes of *D. dadantii* Ech703, *Cedecea neteri* and *Pseudomonas cichorii*. The G + C content was computed in 1Kb sliding windows, with a step of 30 bp, along a genomic region containing the *chs*-like gene. Genes from the variable region are indicated with white arrows. Black arrows show the length and position of the surrounding genes. For each gene, the G + C content at the third codon position (GC3%) is indicated with a black dot. The horizontal line corresponds to the G + C content of the entire region. (PDF 178 kb)
Additional file 12: File S1. Databank of all classified CHS in fasta format. (TXT 980 kb)
Additional file 13: Table S6. Eukaryotic species analyzed in this study. (XLSX 17 kb)
Additional file 14: Table S7. Archeae, Bacteria and Virus genomes analyzed in this study. (XLSX 124 kb)
Additional file 15: Figure S7. Bayesian phylogeny of chitin synthases. (PDF 69 kb)
Additional file 16: File S2. ML phylogeny used to place the root in Fig. 4. (TXT 5 kb)
Additional file 17: Figure S8. Bayesian phylogeny of fungal CHS belonging to division 1. (PDF 189 kb)
Additional file 18: File S3. ML phylogeny used to place the root in Fig. 5. (TXT 6 kb)
Additional file 19: Figure S9. Bayesian phylogeny of fungal CHS belonging to division 2. (PDF 241 kb)
Additional file 20: Figure S10. Bayesian phylogeny of bacterial chitin synthases. (PDF 71 kb)

## Abbreviations

CHS: Chitin synthase(s)
CV: Chlorovirus-like CHS class
ESV: Ectocarpus siliculosus-like CHS class
HAS: Hyaluronan synthase(s)
HGT: Horizontal gene transfer(s)
LBA: Long branch attraction
LOCA: Last Opistokonta common ancestor
ML: Maximum Likelihood
recCHS: Recombined chitin synthase
UNGD: UDP-N-acetylglucosamine 6-dehydrogenase gene
