# Supplementary figures and images for: Genome-wide analyses of chitin synthases identify horizontal gene transfers towards bacteria and allow a robust and unifying classification into fungi
Source: BMC Evol Biol. 2016 Nov 24;16:252. doi: 10.1186/s12862-016-0815-9 (PMC5122149; doi:10.1186/s12862-016-0815-9)

Figure S1

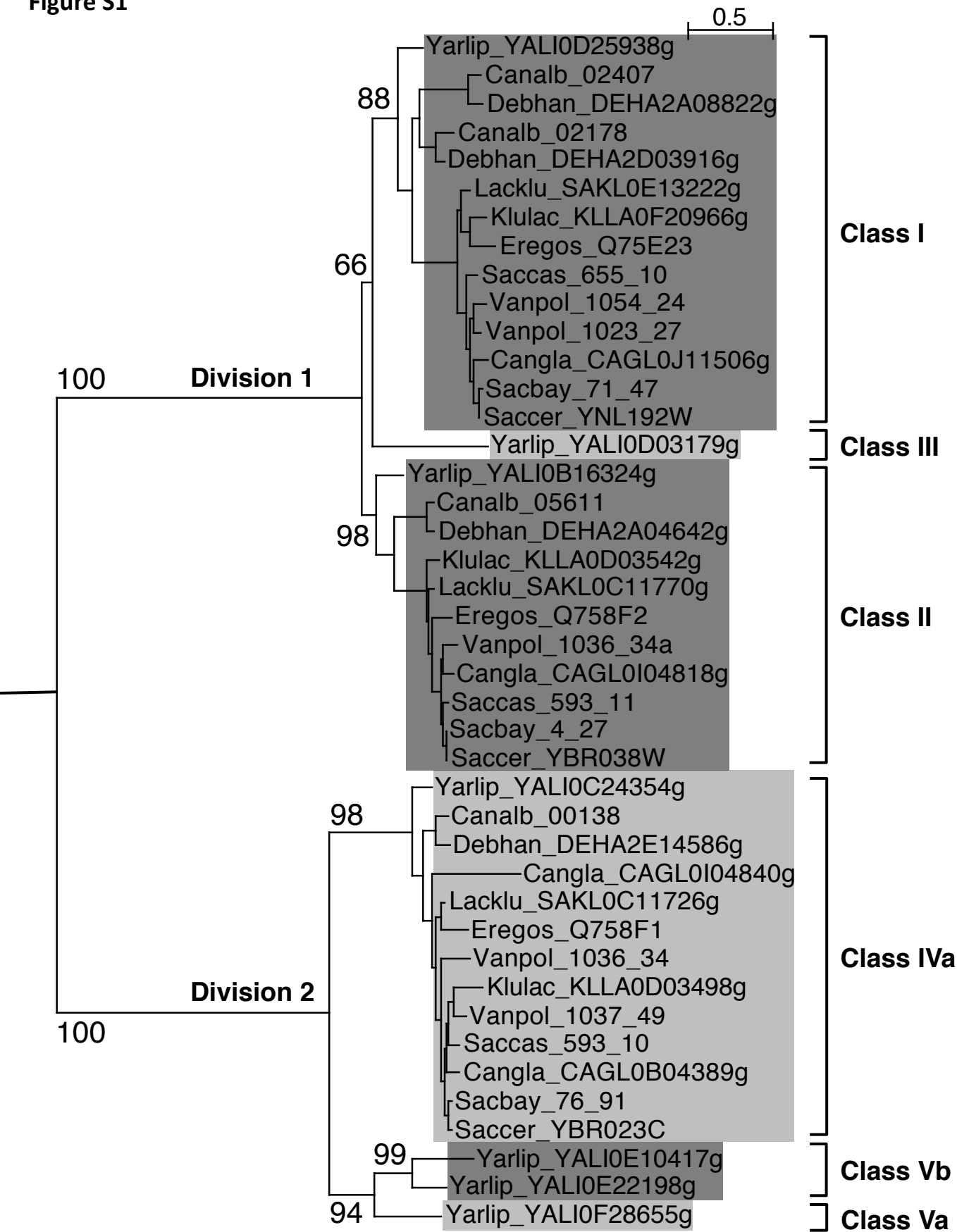

Supplement: Additional file 4: Figure S1. — Phylogeny of Hemiascomycota yeast CHS. A ML phylogeny based on 404 amino acid alignment positions of 42 sequences was constructed with PhyML. Bootstraps of interest and ≥60 are shown above the branches. (PDF 49 kb) [file 12862_2016_815_MOESM4_ESM.pdf]

**Figure S2**

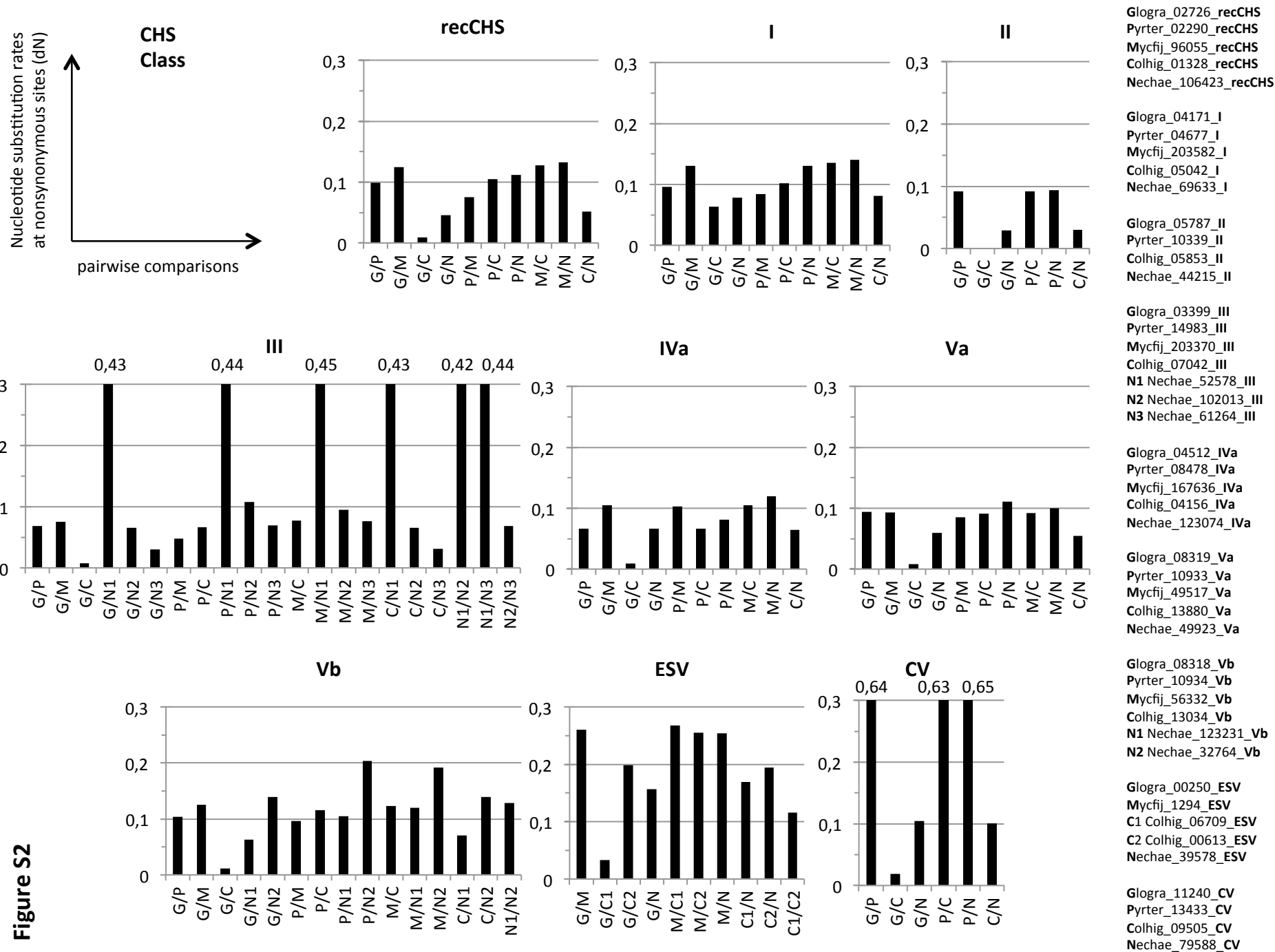

Supplement: Additional file 5: Figure S2. — Nonsynonymous substitution rates (dN) estimated for pairwise comparisons between ascomycota chs from the different classes. Comparisons were restricted to a region spanning the catalytic domain from the c motif to the g motif (Fig. 3 and Additional file 7: Figure S3). The PAML [98] codeml program was used to estimate dN with the codon substitution model of Goldman and Yang [99]. (PDF 47 kb) [file 12862_2016_815_MOESM5_ESM.pdf]

Figure S6

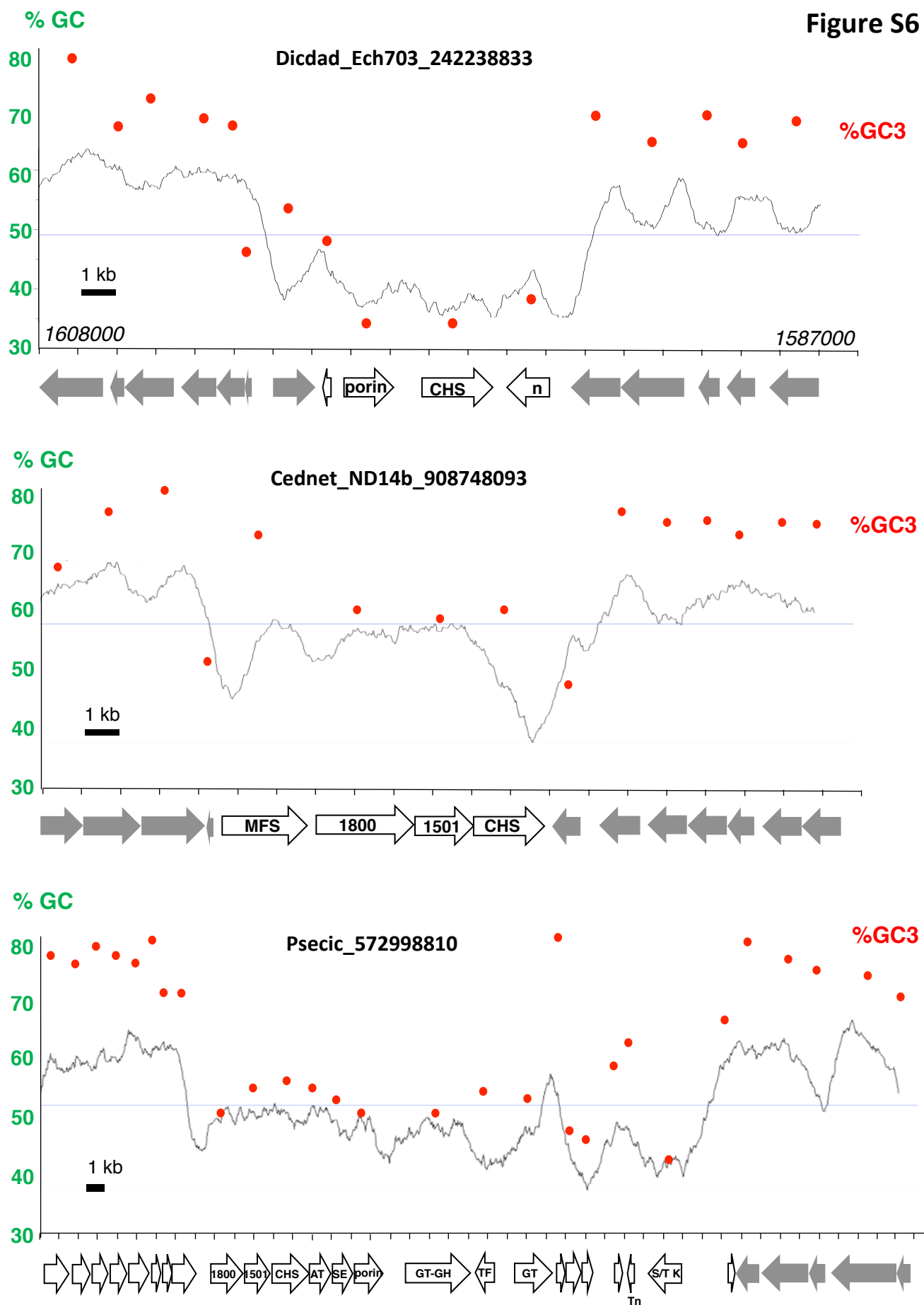

Supplement: Additional file 11: Figure S6. — G + C content variation around the recently laterally transferred chitin synthase genes of D. dadantii Ech703, Cedecea neteri and Pseudomonas cichorii. The G + C content was computed in 1Kb sliding windows, with a step of 30 bp, along a genomic region containing the chs-like gene. Genes from the variable region are indicated with white arrows. Black arrows show the length and position of the surrounding genes. For each gene, the G + C content at the third codon position (GC3%) is indicated with a black dot. The horizontal line corresponds to the G + C content of the entire region. (PDF 178 kb) [file 12862_2016_815_MOESM11_ESM.pdf]

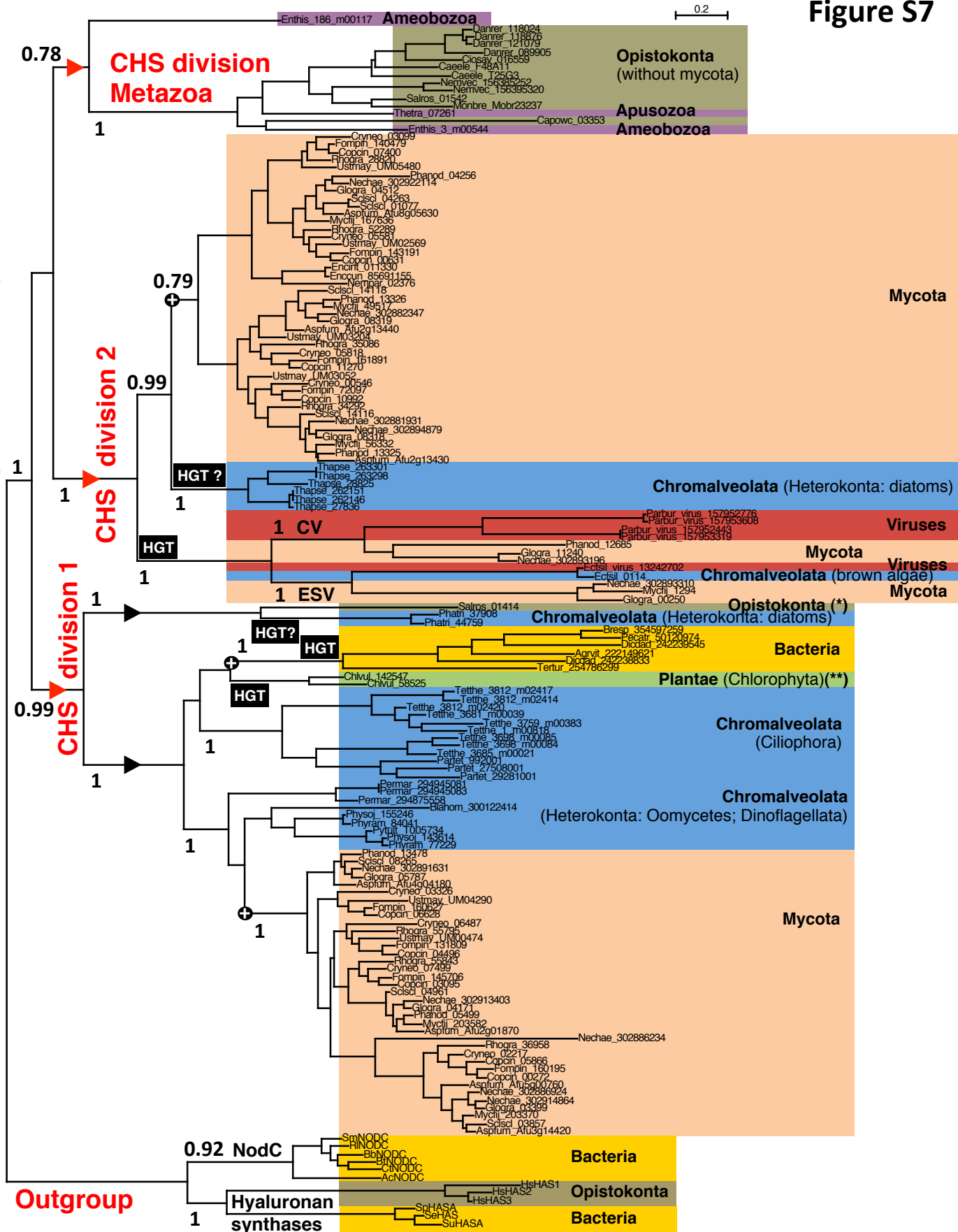

Supplement: Additional file 15: Figure S7. — Bayesian phylogeny of chitin synthases. (PDF 69 kb) [file 12862_2016_815_MOESM15_ESM.pdf]

Figure S8

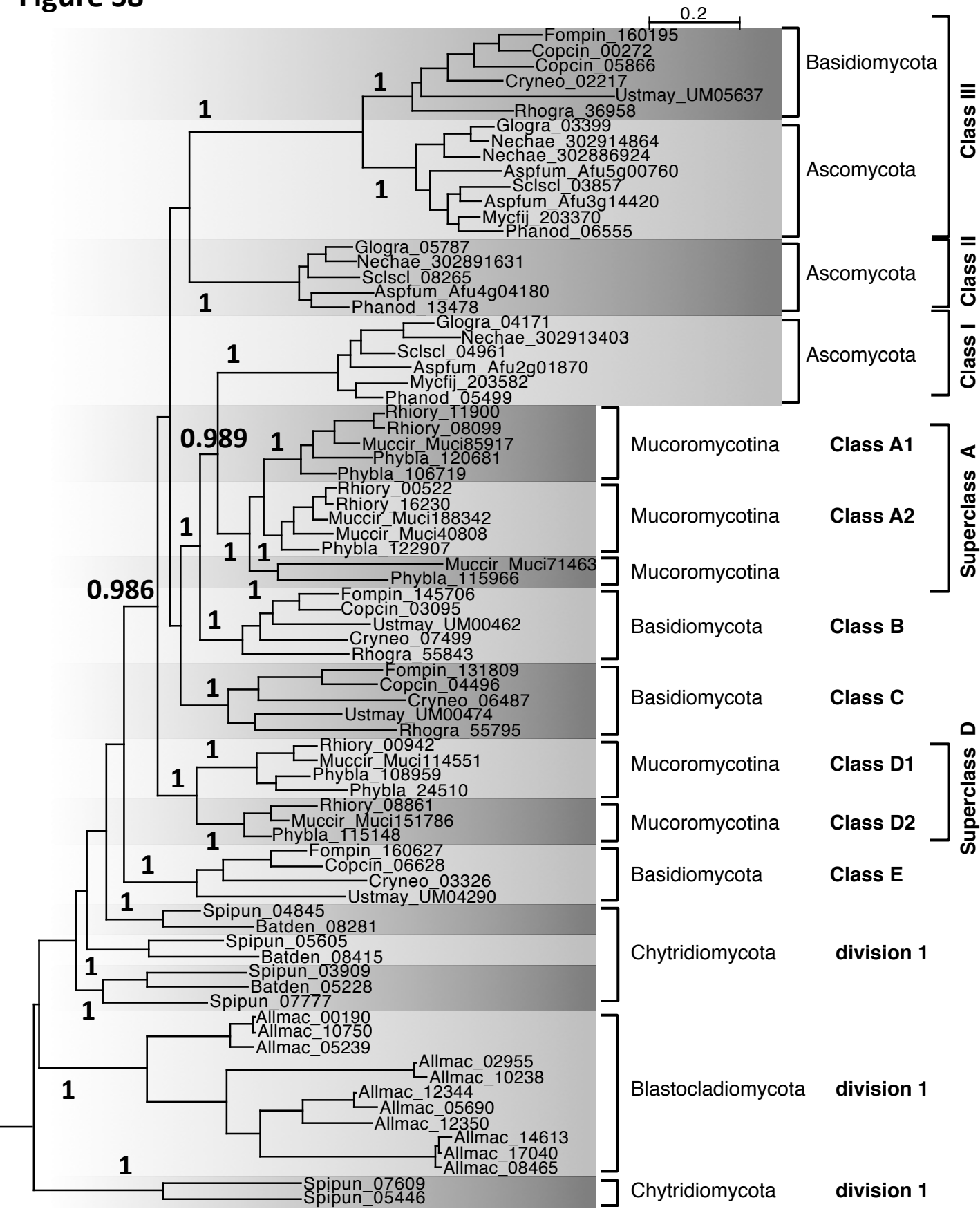

Supplement: Additional file 17: Figure S8. — Bayesian phylogeny of fungal CHS belonging to division 1. (PDF 189 kb) [file 12862_2016_815_MOESM17_ESM.pdf]

**Fig. S9**

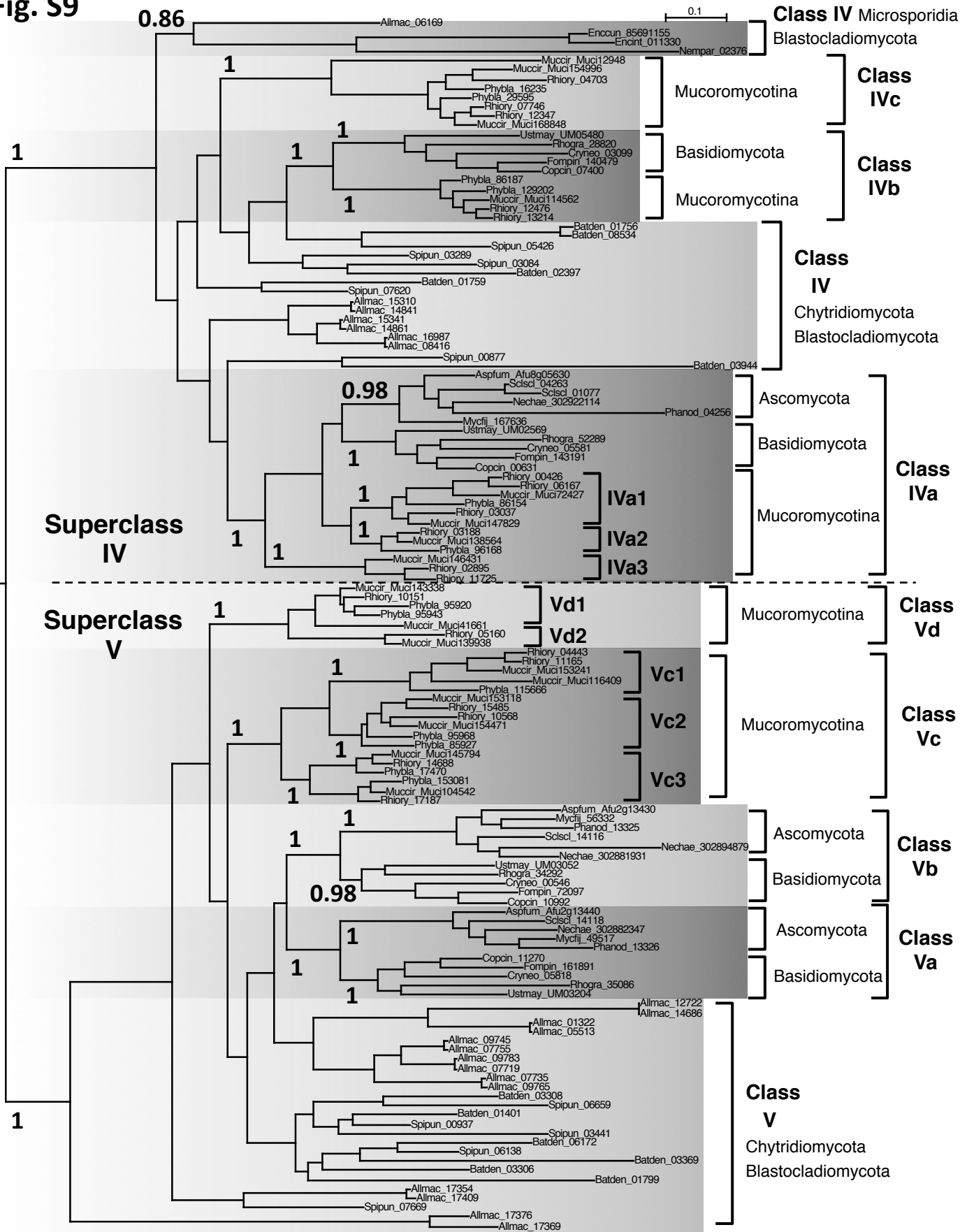

Supplement: Additional file 19: Figure S9. — Bayesian phylogeny of fungal CHS belonging to division 2. (PDF 241 kb) [file 12862_2016_815_MOESM19_ESM.pdf]

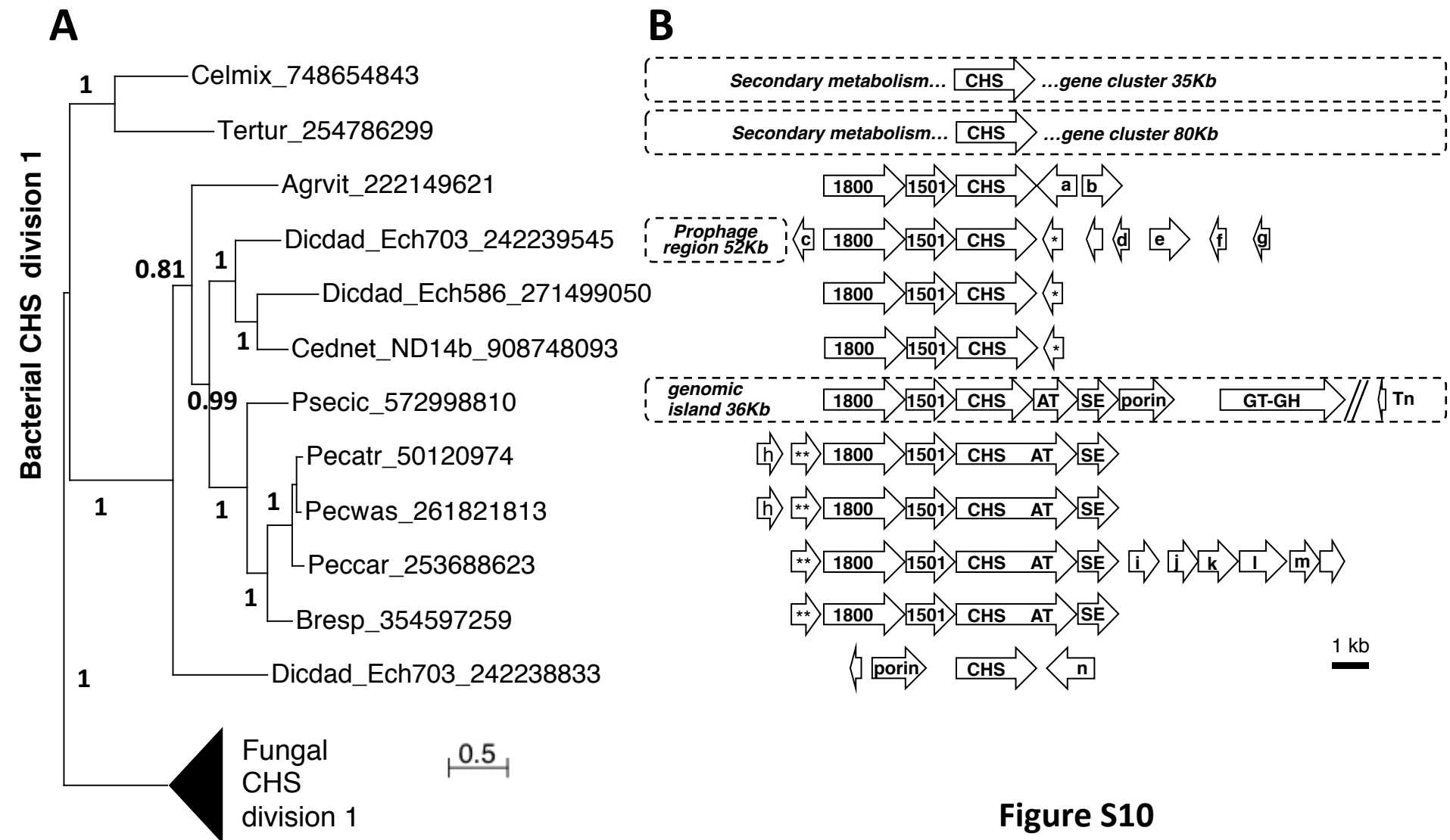

**Figure S10**

Supplement: Additional file 20: Figure S10. — Bayesian phylogeny of bacterial chitin synthases. (PDF 71 kb) [file 12862_2016_815_MOESM20_ESM.pdf]
