# Supplementary material for: Genome-wide analyses of chitin synthases identify horizontal gene transfers towards bacteria and allow a robust and unifying classification into fungi
Source: BMC Evol Biol. 2016 Nov 24;16:252. doi: 10.1186/s12862-016-0815-9 (PMC5122149; doi:10.1186/s12862-016-0815-9)
Supplement: Additional file 7: Figure S3. — Eight conserved signatures in chitin synthases (adapted from [21]). (PDF 90 kb) [file 12862_2016_815_MOESM7_ESM.pdf]

Motifs	a	b	b′	c	d	e	f	g	h
Presumed function	Substrate binding				unknown	binds chitin	catalytic base	binds chitin	unknown
CHS<br>Division 1	T M Y N E<br>Y	D G	K I N S H<br>L	D V G T<br>A	Q N F E Y	L P G	L A E D R I L	Q R R R W	S W G T K<br>T
Bacteria	T L Y N E<br>F	D G	K L D S H	D X G S<br>A T	Q Y X D F<br>Q N<br>F	V P G<br>I<br>L	L A E D R V L<br>Q T I I<br>M	Q R R R W<br>K	S W G T G
Metazoa	T M W H E<br>L Y R	D X	K R W S Q	D G D V<br>A I<br>T	Q X F E Y<br>D	S P G<br>C<br>A	Q G E D R W X<br>M	Q R R R W	X W G X R
Division 2	P C Y T E<br>T A S	D G	K R D S Q	D A D T	Q V Y E Y<br>F	L P G	L G E D R Y L<br>F	Q R R R W	X W G X T
CV	P C Y N E<br>C A R	D G	K R D S L<br>G I	D A D T	Q X X E Y	L P G<br>S	X S E D R X H<br>S	Q Q R R W<br>K	X W G K T
ESV	P A Y S E<br>V N	D G	K K D S L	D A D S<br>G T	Q Q F Q Y<br>D L	L P G	L G T D R R L<br>Q Y	Q R R R W	V W G X S<br>A
recCHS	P C Y K E<br>V R<br>N	D G	K R H C Q	D S D C	Q N F X Y<br>and<br>Q D X E Y	L P G	L G E D R W L	Q R R R W	T W G G P
HAS	A G Y R E<br>P A Q<br>V S N	D G	K R X X M<br>K Q<br>L	D S D T	X S X X Y<br>D<br>A	G P L<br>C	X G D D R H L<br>N E C<br>R	Q Q X R W	X W G T S<br>R

Figure S3. Eight conserved signatures in chitin synthases (adapted from Choquer *et al*., 2004; see also Fig. 3). Motifs a to g have a central position in CHS proteins and are usually surrounded by transmembrane domains. They are predicted to be localized in cytoplasm where the UDP-activated sugar substrate is available. Indeed, their cytoplasmic localization in homologous GT2 glycosyltransferases (hyaluronan synthase, Alginate synthase and NodC) was confirmed with LacZ, PhoA and/or GFP reporter fusions (Heldermon et al., 2001; Karnezis et al., 2003; Oglesby *et al*. 2008; Remminghorst et *al*., 2009; Dorfmueller *et al*., 2014). Recently, the crystal structure from the processive bacterial cellulose synthase BcsA of *Rhodobacter sphaeroides* brought new understanding for the presumed function of these conserved amino acids in polysaccharide synthesis (Morgan *et al*., 2013; Dorfmueller *et al*., 2014). Based on what is proposed for cellulose biosynthesis, one can imagine that motifs a to c could bind to UDP-GlcNAc, motifs e and g could bind to the nascent chitin polysaccharid, and motif f probably acts as the catalytic base. The last conserved motif h is not shared by cellulose synthases but is found in hyaluronan synthases (HAS), NodC activities and alginate synthase Alg8. This motif seem a little appart from the others as it is not found in the CHS core but in the C-terminal region. However, the motif h could interact with the other motifs as its cytoplasmic localization was confirmed by the same studies. Its function is unknown as for the motif d. Substitutions of conserved amino acids by alanine in the catalytic site of the *Saccharomyces cerevisiae* chitin synthase ScCHS2 confirmed that motifs d to h are important or even essential for CHS activity (Nagahashi et al., 1995; Yabe *et al.,* 1998). Duplication of motif d is proposed for recCHS proteins (in black) .
