# Supplementary material for: Genome-wide analyses of chitin synthases identify horizontal gene transfers towards bacteria and allow a robust and unifying classification into fungi
Source: BMC Evol Biol. 2016 Nov 24;16:252. doi: 10.1186/s12862-016-0815-9 (PMC5122149; doi:10.1186/s12862-016-0815-9)
Supplement: Additional file 9: Table S5. — Co-occurrence of DUF1501, DUF1800 and CHS encoding genes in bacterial genomes. (DOCX 60 kb) [file 12862_2016_815_MOESM9_ESM.docx]

| **TableS5 : Co-occurrence of DUF1501, DUF1800 and CHS encoding genes in bacterial genomes** | **#DUF1501** | **#DUF1800** | **#CHS** |
| --- | --- | --- | --- |
| *Proteobacteria; Gammaproteobacteria; Enterobacteriales;* |  |  |  |
| **Brenneria sp. EniD312** | **1** | **1** | **1** |
| Brenneria salicis ATCC15712 | 0 | 0 | 0 |
| Dickeya_dadantii_3937_uid52537 | 0 | 0 | 0 |
| **Dickeya_dadantii_Ech586_uid42519** | **1** | **1** | **1** |
| **Dickeya_dadantii_Ech703_uid59363** | **2** | **2** | **2** |
| Dickeya_zeae_Ech1591_uid59297 | 0 | 0 | 0 |
| **Pectobacterium_atrosepticum_SCRI1043_uid57957** | **1** | **1** | **1** |
| **Pectobacterium_carotovorum_PC1_uid59295** | **1** | **1** | **1** |
| Pectobacterium_carotovorum_subsp._brasiliensis_PBR1692 | 0 | 0 | 0 |
| Pectobacterium_carotovorum_subsp._carotovorum_WPP14 | 0 | 0 | 0 |
| **Pectobacterium_wasabiae_WPP163_uid41297** | **1** | **1** | **1** |
| *Proteobacteria; Gammaproteobacteria; Pseudomonales* |  |  |  |
| **Pseudomonas_cichorii_JBC1** | **1** | **1** | **1** |
| Pseudomonas_syringae_B728a_uid57931 | 0 | 0 | 0 |
| Pseudomonas_syringae_phaseolicola_1448A_uid58099 | 0 | 0 | 0 |
| Pseudomonas_syringae_tomato_DC3000_uid57967 | 0 | 0 | 0 |
| *Proteobacteria; Alphaproteobacteria; Rhizobiales* |  |  |  |
| Agrobacterium_H13_3_uid63403 | 0 | 0 | 0 |
| Agrobacterium_radiobacter_K84_uid58269 | 1 | 1 | 0 |
| Agrobacterium_tumefaciens_C58_uid57865 | 1 | 1 | 0 |
| **Agrobacterium_vitis_S4_uid58249** | **2** | **2** | **1** |
| *Proteobacteria; Gammaproteobacteria; Alteromonadales* |  |  |  |
| **Teredinibacter_turnerae_T7901** | **1** | **1** | **1** |
| Saccharophagus_degradans_2_40 | 1 | 1 | 0 |
