# Supplementary material for: Genome-wide analyses of chitin synthases identify horizontal gene transfers towards bacteria and allow a robust and unifying classification into fungi
Source: BMC Evol Biol. 2016 Nov 24;16:252. doi: 10.1186/s12862-016-0815-9 (PMC5122149; doi:10.1186/s12862-016-0815-9)
Supplement: Additional file 8: Figure S4. — Phylogenies of CHS N- and C-termini regions. The N-terminal region, used for phylogeny, ends before the motif d of the catalytic domain and the C-terminal region starts after this motif (Fig. 3 and Additional file 7: Figure S3) The two corresponding alignments were obtained by extracting these regions from the alignement obtained with the full sequences. They were then filtered with BMGE prior to the tree reconstruction analysis. This resulted in 72 amino acid alignment positions of 119 CHS N-terminal region sequences and 148 amino acid alignment positions of 120 CHS C-terminal region sequences. Phylogenetic trees were constructed with PhyML (A-B) and MrBayes (C-D) Bootstraps ≥60 (PhyML) and posterior probabilities (MrBayes) of interest are shown above the branches. (PDF 133 kb) [file 12862_2016_815_MOESM8_ESM.pdf]

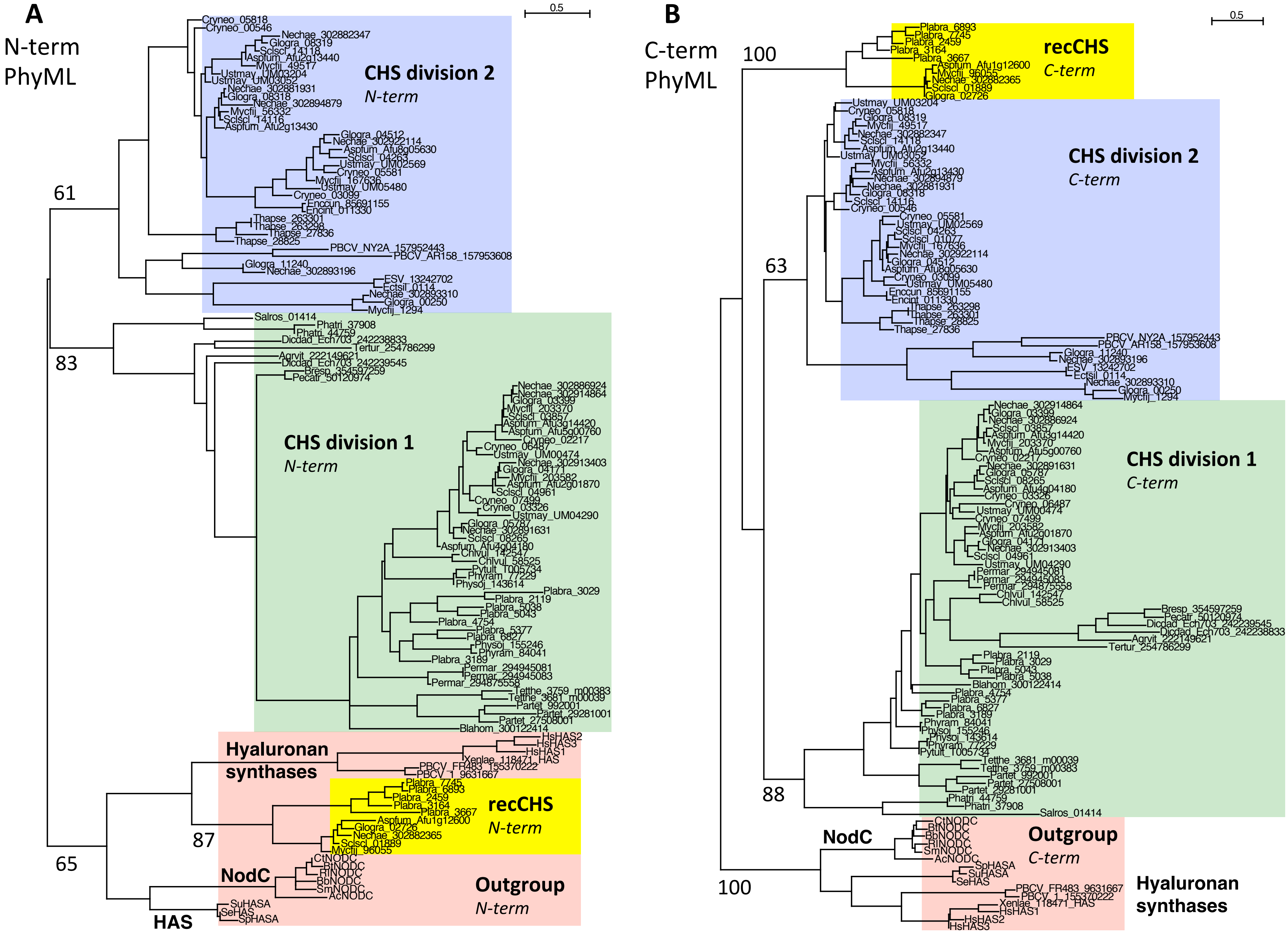

A
N-term
PhyML
0.5
CHS division 2
N-term
Cryneo_05818
Cryneo_00546
Nechae_302882347
Glogra_08319
Sclscl_14118
Aspfum_Afu2g13440
Mycfij_49517
Ustmay_UM03204
Ustmay_UM03052
Nechae_302881931
Glogra_08318
Nechae_302894879
Mycfij_56332
Sclscl_14116
Aspfum_Afu2g13430
Glogra_04512
Nechae_302922114
Aspfum_Afu8g05630
Sclscl_04263
Ustmay_UM02569
Cryneo_05581
Mycfij_167636
Ustmay_UM05480
Cryneo_03099
Enccun_85691155
Encint_011330
Thapse_263301
Thapse_263298
Thapse_27836
Thapse_28825
PBCV_NY2A_157952443
PBCV_AR158_157953608
Glogra_11240
Nechae_302893196
ESV_13242702
Ectsil_0114
Nechae_302893310
Glogra_00250
Mycfij_1294
61
83
CHS division 1
N-term
Salros_01414
Phatri_37908
Phatri_44759
Dicdad_Ech703_242238833
Tertur_254786299
Agrvit_222149621
Dicdad_Ech703_242239545
Bresp_354597259
Pecatr_50120974
Nechae_302886924
Nechae_302914864
Glogra_03399
Mycfij_203370
Sclscl_03857
Aspfum_Afu3g14420
Aspfum_Afu5g00760
Cryneo_02217
Cryneo_06487
Ustmay_UM00474
Nechae_302913403
Glogra_04171
Mycfij_203582
Aspfum_Afu2g01870
Sclscl_04961
Cryneo_07499
Cryneo_03326
Ustmay_UM04290
Glogra_05787
Nechae_302891631
Sclscl_08265
Aspfum_Afu4g04180
Chlvul_142547
Chlvul_58525
Pytult_T005734
Phyram_77229
Physoj_143614
Plabra_3029
Plabra_2119
Plabra_5038
Plabra_5043
Plabra_4754
Plabra_5377
Plabra_6827
Physoj_155246
Phyram_84041
Plabra_3189
Permar_294945081
Permar_294945083
Permar_294875558
Tetthe_3759_m00383
Tetthe_3681_m00039
Partet_992001
Partet_29281001
Partet_27508001
Blahom_300122414
Hyaluronan
synthases
HsHAS2
HsHAS3
HsHAS1
Xenlae_118471_HAS
PBCV_FR483_155370222
PBCV_1_9631667
recCHS
N-term
Plabra_7745
Plabra_6893
Plabra_2459
Plabra_3164
Plabra_3667
Aspfum_Afu1g12600
Glogra_02726
Nechae_302882365
Sclscl_01889
Mycfij_96055
87
65
NodC
CtNODC
BtNODC
RlNODC
BbNODC
SmNODC
AcNODC
Outgroup
N-term
SuHASA
SeHAS
SpHASA
HAS
B
C-term
PhyML
0.5
100
recCHS
C-term
Plabra_6893
Plabra_7745
Plabra_2459
Plabra_3164
Plabra_3667
Aspfum_Afu1g12600
Mycfij_96055
Nechae_302882365
Sclscl_01889
Glogra_02726
CHS division 2
C-term
Ustmay_UM03204
Cryneo_05818
Glogra_08319
Mycfij_49517
Nechae_302882347
Sclscl_14118
Aspfum_Afu2g13440
Ustmay_UM03052
Mycfij_56332
Aspfum_Afu2g13430
Nechae_302894879
Nechae_302881931
Glogra_08318
Sclscl_14116
Cryneo_00546
Cryneo_05581
Ustmay_UM02569
Sclscl_04263
Sclscl_01077
Mycfij_167636
Nechae_302922114
Glogra_04512
Aspfum_Afu8g05630
Cryneo_03099
Ustmay_UM05480
Enccun_85691155
Encint_011330
Thapse_263298
Thapse_263301
Thapse_28825
Thapse_27836
63
PBCV_NY2A_157952443
PBCV_AR158_157953608
Glogra_11240
Nechae_302893196
ESV_13242702
Ectsil_0114
Nechae_302893310
Glogra_00250
Mycfij_1294
CHS division 1
C-term
Nechae_302914864
Glogra_03399
Nechae_302886924
Sclscl_03857
Aspfum_Afu3g14420
Mycfij_203370
Aspfum_Afu5g00760
Cryneo_02217
Nechae_302891631
Glogra_05787
Sclscl_08265
Aspfum_Afu4g04180
Cryneo_03326
Cryneo_06487
Ustmay_UM00474
Cryneo_07499
Mycfij_203582
Aspfum_Afu2g01870
Glogra_04171
Nechae_302913403
Sclscl_04961
Ustmay_UM04290
Permar_294945081
Permar_294945083
Permar_294875558
Chlvul_142547
Chlvul_58525
Bresp_354597259
Pecatr_50120974
Dicdad_Ech703_242239545
Dicdad_Ech703_242238833
Agrvit_222149621
Tertur_254786299
Plabra_2119
Plabra_3029
Plabra_5043
Plabra_5038
Blahom_300122414
Plabra_4754
Plabra_5377
Plabra_6827
Plabra_3189
Phyram_84041
Physoj_155246
Physoj_143614
Phyram_77229
Pytult_T005734
Tetthe_3681_m00039
Tetthe_3759_m00383
Partet_992001
Partet_27508001
Partet_29281001
Phatri_44759
Phatri_37908
Salros_01414
88
NodC
CtNODC
BtNODC
BbNODC
RlNODC
SmNODC
AcNODC
Outgroup
C-term
SpHASA
SuHASA
SeHAS
PBCV_FR483_9631667
PBCV_1_155370222
Xenlae_118471_HAS
HsHAS1
HsHAS2
HsHAS3
Hyaluronan
synthases
100

Figure S4

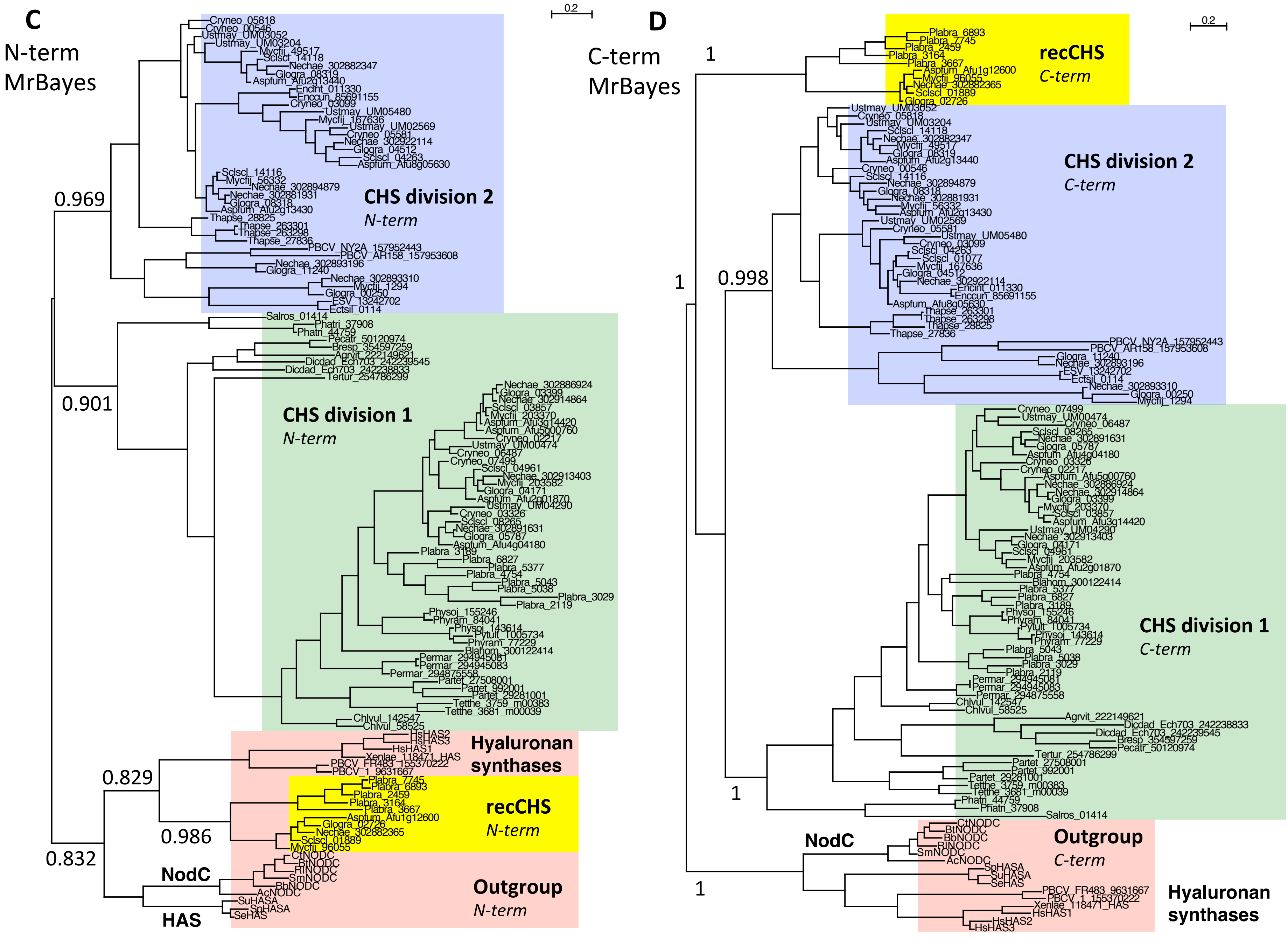
C
N-term
MrBayes
0.2
Cryneo_05818
Cryneo_00546
Ustmay_UM03052
Ustmay_UM03204
Mycfij_49517
Sclscl_14118
Nechae_302882347
Glogra_08319
Aspfum_Afu2g13440
Encint_011330
Enccun_85691155
Cryneo_03099
Ustmay_UM05480
Mycfij_167636
Ustmay_UM02569
Cryneo_05581
Nechae_302922114
Glogra_04512
Sclscl_04263
Aspfum_Afu8g05630
Sclscl_14116
Mycfij_56332
Nechae_302894879
Nechae_302881931
Glogra_08318
Aspfum_Afu2g13430
Thapse_28825
Thapse_263301
Thapse_263298
Thapse_27836
PBCV_NY2A_157952443
PBCV_AR158_157953608
Nechae_302893196
Glogra_11240
Nechae_302893310
Mycfij_1294
Glogra_00250
ESV_13242702
Ectsil_0114
CHS division 2
N-term
0.969
Salros_01414
Phatri_37908
Phatri_44759
Pecatr_50120974
Bresp_354597259
Agrvit_222149621
Dicdad_Ech703_242239545
Dicdad_Ech703_242238833
Tertur_254786299
0.901
CHS division 1
N-term
Nechae_302886924
Glogra_03399
Nechae_302914864
Sclscl_03857
Mycfij_203370
Aspfum_Afu3g14420
Aspfum_Afu5g00760
Cryneo_02217
Ustmay_UM00474
Cryneo_06487
Cryneo_07499
Sclscl_04961
Nechae_302913403
Mycfij_203582
Glogra_04171
Aspfum_Afu2g01870
Ustmay_UM04290
Cryneo_03326
Sclscl_08265
Nechae_302891631
Glogra_05787
Aspfum_Afu4g04180
Plabra_3189
Plabra_6827
Plabra_5377
Plabra_4754
Plabra_5043
Plabra_5038
Plabra_3029
Plabra_2119
Physoj_155246
Phyram_84041
Physoj_143614
Pytult_1005734
Phyram_77229
Blahom_300122414
Permar_294945081
Permar_294945083
Permar_294875558
Partet_27508001
Partet_992001
Partet_29281001
Tetthe_3759_m00383
Tetthe_3681_m00039
Chlvul_142547
Chlvul_58525
HsHAS2
HsHAS3
HsHAS1
Xenlae_118471_HAS
PBCV_FR483_155370222
PBCV_1_9631667
Hyaluronan
synthases
0.829
Plabra_7745
Plabra_6893
Plabra_2459
Plabra_3164
Plabra_3667
Aspfum_Afu1g12600
Glogra_02726
Nechae_302882365
Sclscl_01889
Mycfij_96055
recCHS
N-term
0.986
CtNODC
BtNODC
RlNODC
SmNODC
BbNODC
AcNODC
SuHASA
SpHASA
SeHAS
0.832
NodC
HAS
Outgroup
N-term
D
C-term
MrBayes
0.2
Plabra_6893
Plabra_7745
Plabra_2459
Plabra_3164
Plabra_3667
Aspfum_Afu1g12600
Mycfij_96055
Nechae_302882365
Sclscl_01889
Glogra_02726
recCHS
C-term
1
Ustmay_UM03052
Cryneo_05818
Ustmay_UM03204
Sclscl_14118
Nechae_302882347
Mycfij_49517
Glogra_08319
Aspfum_Afu2g13440
Cryneo_00546
Sclscl_14116
Nechae_302894879
Glogra_08318
Nechae_302881931
Mycfij_56332
Aspfum_Afu2g13430
Ustmay_UM02569
Cryneo_05581
Ustmay_UM05480
Cryneo_03099
Sclscl_04263
Sclscl_01077
Mycfij_167636
Glogra_04512
Nechae_302922114
Encint_011330
Enccun_85691155
Aspfum_Afu8g05630
Thapse_263301
Thapse_263298
Thapse_28825
Thapse_27836
PBCV_NY2A_157952443
PBCV_AR158_157953608
Glogra_11240
Nechae_302893196
ESV_13242702
Ectsil_0114
Nechae_302893310
Glogra_00250
Mycfij_1294
CHS division 2
C-term
0.998
1
Cryneo_07499
Ustmay_UM00474
Cryneo_06487
Sclscl_08265
Nechae_302891631
Glogra_05787
Aspfum_Afu4g04180
Cryneo_03326
Cryneo_02217
Aspfum_Afu5g00760
Nechae_302886924
Nechae_302914864
Glogra_03399
Mycfij_203370
Sclscl_03857
Aspfum_Afu3g14420
Ustmay_UM04290
Nechae_302913403
Glogra_04171
Sclscl_04961
Mycfij_203582
Aspfum_Afu2g01870
Plabra_4754
Blahom_300122414
Plabra_5377
Plabra_6827
Plabra_3189
Physoj_155246
Phyram_84041
Pytult_1005734
Physoj_143614
Phyram_77229
Plabra_5043
Plabra_5038
Plabra_3029
Plabra_2119
Permar_294945081
Permar_294945083
Permar_294875558
Chlvul_142547
Chlvul_58525
Agrvit_222149621
Dicdad_Ech703_242238833
Dicdad_Ech703_242239545
Bresp_354597259
Pecatr_50120974
Tertur_254786299
Partet_27508001
Partet_992001
Partet_29281001
Tetthe_3759_m00383
Tetthe_3681_m00039
Phatri_44759
Phatri_37908
Salros_01414
CHS division 1
C-term
1
CtNODC
BtNODC
BbNODC
RlNODC
SmNODC
AcNODC
SpHASA
SuHASA
SeHAS
PBCV_FR483_9631667
PBCV_1_155370222
Xenlae_118471_HAS
HsHAS1
HsHAS2
HsHAS3
NodC
Outgroup
C-term
Hyaluronan
synthases
1

Figure S4
