# Supplementary material for: Genome-wide analyses of chitin synthases identify horizontal gene transfers towards bacteria and allow a robust and unifying classification into fungi
Source: BMC Evol Biol. 2016 Nov 24;16:252. doi: 10.1186/s12862-016-0815-9 (PMC5122149; doi:10.1186/s12862-016-0815-9)
Supplement: Additional file 10: Figure S5. — Phylogenies of DUF1800 and DUF1501 bearing proteins. ML phylogenies based on (A) 383 amino acid alignment positions of 14 DUF1800 bearing sequences and (B) 335 amino acid alignment positions of 14 DUF1501 bearing sequences were constructed with PhyML. Bootstraps of interest and ≥60 are shown above the branches. (PDF 51 kb) [file 12862_2016_815_MOESM10_ESM.pdf]

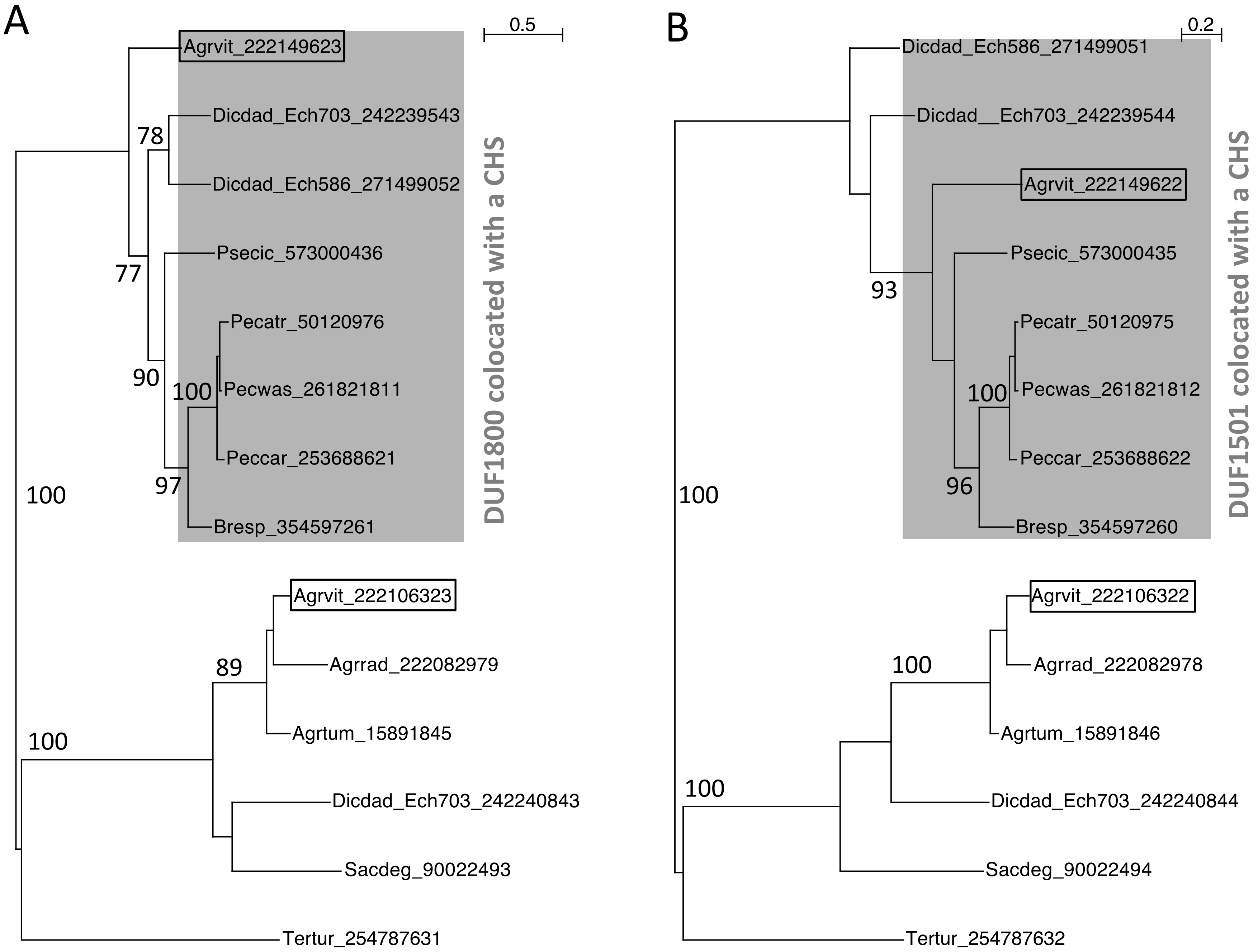
A
0.5
Agrvit_222149623
Dicdad_Ech703_242239543
Dicdad_Ech586_271499052
Psecic_573000436
Pecatr_50120976
Pecwas_261821811
Peccar_253688621
Bresp_354597261
78
77
90
100
97
100
DUF1800 colocated with a CHS
Agrvit_222106323
Agrrad_222082979
Agrtum_15891845
Dicdad_Ech703_242240843
Sacdeg_90022493
Tertur_254787631
89
100
B
0.2
Dicdad_Ech586_271499051
Dicdad__Ech703_242239544
Agrvit_222149622
Psecic_573000435
Pecatr_50120975
Pecwas_261821812
Peccar_253688622
Bresp_354597260
93
100
96
100
DUF1501 colocated with a CHS
Agrvit_222106322
Agrrad_222082978
Agrtum_15891846
Dicdad_Ech703_242240844
Sacdeg_90022494
Tertur_254787632
100
100

Figure S5
